# Robust bilinear rotations

**DOI:** 10.1126/sciadv.adx7094

**Published:** 2025-08-29

**Authors:** Yannik T. Woordes, Tony Reinsperger, Sebastian Ehni, Burkhard Luy

**Affiliations:** ^1^ Institute of Organic Chemistry and Institute for Biological Interfaces 4 - Magnetic Resonance, Karlsruhe Institute of Technology (KIT), Hermann-von-Helmholtz-Platz 1, 76344 Eggenstein-Leopoldshafen, Germany.; ^2^ Clinical NMR Method Development, Biopharma and Applied Division (BP&A), Bruker BioSpin GmbH & Co. KG, Rudolf-Planck-Str. 23, 76275 Ettlingen, Germany.; ^3^ Bruker Switzerland AG, Industriestr. 26, 8117 Fällanden, Switzerland.

## Abstract

Bilinear rotation elements allow interaction-dependent manipulations of spins in quantum technologies and, particularly, in spectroscopy. We used bilinear rotation in nuclear magnetic resonance (NMR) spectroscopy and derived several ways of introducing robustness into the filter element. We distinguished two performance levels: the coupling dependent inversion of polarization and the full bilinear π-rotation capability. In addition, all four essential variants of bilinear π rotations—BIRD*^d^*, BIRD*^r^*, BIRD^*d*,*X*^, and BIRD^*r*,*X*^ filters—were given for all cases studied. In the first step, adiabatic CHIRP-type and BUBI/BUBU pulses lead to improved robustness with respect to offset/detuning effects and variations in *B*_1_-field strengths. In the second step, we optimized time-optimal coupling–compensated BIRD elements. Together with correspondingly optimized pulse shapes, we established fully coupling, offset, and *B*_1_-compensated bilinear π-rotation (COB-BIRD) elements and characterized them in theory and experiment. Overall, this demonstrated the use of the robust bilinear rotation capability on a partially aligned sample in a homodecoupled two-dimensional NMR experiment.

## Teaser

A bilinear rotation element compensated for offset and coupling enhances applications in NMR and other quantum technologies.

## INTRODUCTION

The specific manipulation of spins is essential for magnetic resonance and other fields of quantum technology such as modern spintronics or quantum computing. Decoupling from the environment (*1*) as well as decoupling from other qubits can be achieved physically (*2*) and via corresponding bilinear spin manipulations (*3*) to allow for relaxation compensation and quantum error correction as long as robust interaction-dependent bilinear rotations are available in corresponding spin systems. However, the main field of interest so far is modern nuclear magnetic resonance (NMR) spectroscopy, where bilinear rotations have been originally developed and driven into a bouquet of highly useful basic elements for a multitude of applications. Thereby, depending on the presence or absence of specific interspin couplings, different spin rotations are performed. Currently, such bilinear rotations are, with very few exceptions, typically implemented using simple hard pulses and therefore do not allow the application to broader ranges of effective bilinear coupling constants and/or spectral ranges. Using the first bilinear rotation, bilinear rotation decoupling (BIRD), as the example case in the following, several ways of improving the robustness of bilinear rotation elements are derived and characterized systematically in both theory and demonstration experiments.

BIRD was introduced by Garbow *et al.* (*4*), and its application width was highly enhanced by the systematic generalization of the approach a decade later by Kövér and colleagues (*5*). BIRD filters laid the basis for a large number of important spectral improvements in NMR spectroscopy. Next to its original application in homonuclear decoupling, for which today many slightly more sophisticated approaches form a major part of pure shift spectroscopy (*6*–*15*), it has been used for spectral cleanup (*16*, *17*), indirect decoupling (*18*, *19*), and the measurement of a large variety of different couplings (*20*–*24*).

The BIRD element has been generalized in many ways. It has been extended to bilinear rotations other than π (*25*–*28*), combined with pulsed field gradients (*29*, *30*), adjusted to accommodate exchanging protons (*21*, *31*), and many more. Recent developments for bilinear rotations include the extension to uniformly isotope-labeled samples with BASEREX [BAnd SElective REfocusing on the X nucleus (*32*–*34*)] and the NORD (NO Relaxation Delay) element for the acquisition of nested experiments in so-called supersequences (*35*–*37*).

All enumerated pulse sequence elements have been developed based on the simple BIRD element, which works well only for a small range of heteronuclear coupling constants, which, together with the sometimes unwanted evolution of homonuclear couplings, may be considered the main drawback of current bilinear rotations. Already in its classical application, the isotope selective inversion of carbon-bound protons, the occurrence of sp-, sp^2^-, and sp^3^-hybridized carbons leads to a range of ^1^*J*_CH_ coupling constants of 120 to 250 Hz, which cannot be covered satisfyingly with the simple element. The Pines group was already aware of this in the original BIRD publication (*4*), developing what they called the *J*-compensated BIRD (JC-BIRD) element. However, as presented in the following sections, the element does not fulfill all requirements of real *J* compensation.

A second, important drawback of BIRD elements is the strong variation of performance with respect to large spectral ranges, i.e., the offset or detuning ranges, as well as with variations of *B*_1_ fields, leading to varying radio frequency (rf) amplitudes or Rabi frequencies, respectively. Looking at various options for obtaining offset, *B*_1_, and coupling-compensated BIRD elements, we developed several pulse sequences, which are all improvements compared to the simple hard-pulse BIRD. In the following sections, we first characterize the properties of the original four variants BIRD*^d^*, BIRD*^r^*, BIRD^*d*,*X*^, and BIRD^*r*,*X*^ that lead to inversions of four different spin classes as defined later, as well as the initial JC-BIRD in detail. We then look into easily applied enhancement of robustness with respect to wide offset ranges on the *X* nucleus, demonstrated by several versions of an adiabatic BIRD and BUBI/BUBU-BIRD with optimal control–derived broadband universal rotation and inversion pulses. Subsequently, coupling compensation for a bilinear π-rotation element is systematically derived, and, last, full compensation is achieved by the application of additional sets of optimal control–derived shaped pulses with uncommon flip angles. The resulting Coupling, Offset, and B_1_-inhomogeneity compensated sequence is called COB-BIRD following the nomenclature for the first systematically compensated element, the COB-INEPT (*38*, *39*). Examples for all classes of BIRD elements are characterized in detail in theory and experiment.

## RESULTS

### Original BIRD variants, JC-BIRD, and their rotational performance

**Fig. 1.**
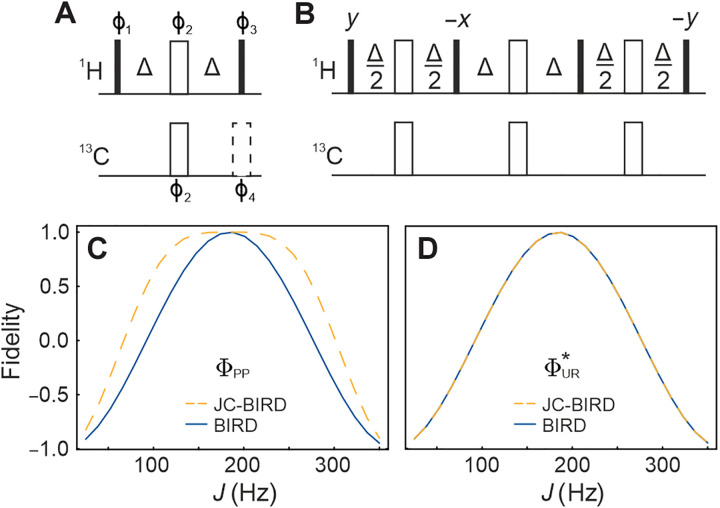
Theoretical comparison of the BIRD and JC-BIRD pulse sequences on their efficacy of spin inversion and universal rotation to a variation in *J* coupling. Original BIRD (**A**) and JC-BIRD (**B**) pulse sequences (*4*, *5*) and their robustness against *J* coupling variations (**C** and **D**). Solid and open rectangles correspond to 90° and 180° pulses, respectively; phases are *x* unless indicated otherwise, and transfer delays are calibrated to match Δ = 1/(2 × ^1^*J*_CH_). The phases ϕ_1_ to ϕ_4_ of the BIRD sequence (A) can be manipulated according to Table 1 to obtain the four possible effective bilinear rotations. If ϕ_4_ is not indicated, the dashed pulse is left out. The $\Phi_{\text{PP}}$ (C) and $\Phi_{\text{UR}}^{*}$ (D) fidelities of both the BIRD^*d*,*X*^ element (solid blue, ϕ_3_ = *x*, no dashed 180° pulse applied) and the JC-BIRD^*d*,*X*^ element (dashed orange) are simulated using on-resonant hard pulses and Δ = 1/(2 × 185 Hz).

The original BIRD filter was introduced as a means to use the ^13^C spin (or other heteronuclei) as a local decoupler field to achieve homonuclear ^1^H decoupling (*4*). This manipulation on a spin system can be described as a bilinear π rotation on a heteronuclear two-spin system, hence, the acronym BIRD. With both spins on resonance, i.e., neglecting offsets/detuning, the heteronuclear coupling Hamiltonian sandwiched between two 90° pulses (Fig. 1A) yields a propagator of the form

$$U_{H^{d}}=\text{exp}\left(-i\pi 2I_{\alpha }^{d}S_{\beta }\right)$$ (1)

with α, β∈{x,y,z} and $I^{d}$ representing ^1^H magnetization of spins bound to the heteronucleus *S* using the conventional letters for the description of spin and spin system operators. A deviating annotation, the *d*,*r*,*X* nomenclature introduced in (*5*), is used to describe the three different spin classes of interest, where the heteronucleus (*X*) as well as directly bound (*d*; protons directly attached to the heteronucleus *X*) and remote protons (*r*; all other protons) are distinguished. However, the flip angle only corresponds to 180° if the BIRD delays are matched to the value of the heteronuclear coupling (see Fig. 1). In the case of delay, mismatch magnetization dissipates, which causes sensitivity losses. This matter was seemingly addressed in the original publication (*4*), and the resulting JC-BIRD pulse sequence is depicted in Fig. 1 along with the basic BIRD filter that allows for various modifications (*5*), which is discussed in the following.

The fidelity of any of these pulse sequences can be assessed just like any 180° rotation. Depending on the particular application of interest, either only the selective inversion performance with respect to *z* magnetization is sufficient or the full universal bilinear rotation properties are needed. An example for the first case is the application of BIRD as a simple isotope filter, whereas applications during *t*_1_-evolution periods with transverse magnetization evolving would be an example for the latter. The degree of proton spin inversion for a BIRD*^d^* filter can be measured by a quality factor designed for point-to-point transfer according to

$$\begin{array}{c} \Phi_{\text{PP}}\left(I_{z}^{d}\to -I_{z}^{d}\right)=\langle \rho_{T}\;|\;\rho_{N}\rangle =\\ \text{Tr}\left[\left(-I_{z}^{d}\right)U_{N}\cdots U_{j}\cdots U_{1}I_{z}^{d}U_{1}^{†}\cdots U_{j}^{†}\cdots U_{N}^{†}\right] \end{array}$$ (2)

with the starting spin density operator $\rho_{0}=I_{z}^{d}$ , target spin density operator $\rho_{T}=-I_{z}^{d}$ , and all propagators *U* that comprise all relevant spins including, as a minimum, all directly coupled protons and the heteronucleus *S*. For simplicity, we consider a simple heteronuclear two-spin system here, which is sufficient for the evaluation of sequences. The quality factor $\Phi_{\text{PP}}$ may adopt values in the range of −1 to 1. A corresponding quality factor for universal (bilinear) rotation properties of a BIRD*^d^* filter with the same range of values is given by

$$\begin{array}{c} \Phi_{\text{UR}}=ℜ\langle U_{T}\;|\;U_{\text{eff}}\rangle =ℜ\left[\text{Tr}\left(U_{H^{d}}^{†}U_{N}\cdots U_{j}\cdots U_{1}\right)\right] \end{array}$$ (3)

where the target propagator $U_{T}=U_{H^{d}}$ , and the matrix scalar product is considered to be normalized. The cost function is sensitive to a phase factor $e^{i\phi }$ in the target operator, where, for a two-spin system, the phase may adopt values of ±1 or ±*i*. This sensitivity is needed for optimizations, as has been discussed for single-spin optimizations in detail in (*40*), and is used later. For an evaluation of bilinear rotations, instead, a cost function insensitive to phase factors and with a range equaling that of $\Phi_{\text{PP}}$ seems more appropriate. Such a cost is given by

$$\begin{array}{c} \Phi_{\text{UR}}^{*}=2{\langle U_{T}\;|\;U_{\text{eff}}\rangle }^{2}-1=\\ 2{\left[\text{Tr}\left(U_{H^{d}}^{†}U_{N}\cdots U_{j}\cdots U_{1}\right)\right]}^{2}-1 \end{array}$$ (4)

The complex square ensures cancelation of phase factors, and the normalized matrix scalar product as well as the other additions guarantee a range from −1 to 1 also for the full bilinear rotation. A third cost function $\Phi_{\text{UR}\left({}^{1}H\right)}^{*}$ evaluating just the ^1^H contribution to the target propagator is introduced in the Supplementary Materials.

A full list of target functions for all phase settings for ϕ_1_ to ϕ_4_ is given in the Supplementary Materials. For obtaining all four possible BIRD variants, only a subset with ϕ_1_ = ϕ_2_ = *x* and varying ϕ_3_ and ϕ_4_ is sufficient, for which the corresponding bilinear testing operators $U_{H^{d}}$ and corresponding *J* = 0 operators $U_{H^{r}}$ are shown in Table 1. Required performances for *J* = 0 Hz do not involve any coupling evolution and are simply defined by pulses applied to the individual spins. Corresponding operations as listed in Table 1 are therefore easily achieved for all offsets, where also $\Phi_{\text{UR}}^{*}$ with $U_{H^{d}}$ shows good performance.

**Table 1.** Effective rotation axes and phase factors of the bilinear universal rotation for each essential BIRD element. BIRD rotations with their effective target propagators acting on directly and remotely bound protons ( $U_{H^{d}}$ and $U_{H^{r}}$ , respectively) together with their phase factors $e^{i\phi }$ . ϕ_1_ and ϕ_2_ of Fig. 1 are *x* for all four cases. Additional phase combinations can be found in table S1.

ϕ_3_	ϕ_4_	$U_{H^{r}}\left(J=0\right)$	$e^{i\phi }$	$U_{H^{d}}$	$e^{i\phi }$	Descriptor
*x*	-	$\text{exp}\left(i\pi S_{x}\right)$	−1	$\text{exp}\left(i\pi 2I_{y}S_{y}\right)$	1	*d*,*X*
*x*	*x*	1	1	$\text{exp}\left(i\pi 2I_{y}S_{z}\right)$	1	*d*
−*x*	-	$\text{exp}\left(i\pi 2I_{x}S_{x}\right)$	−*i*	$\text{exp}\left(i\pi 2I_{z}S_{y}\right)$	−1	*r*,*X*
−*x*	*x*	$\text{exp}\left(i\pi I_{x}\right)$	−1	$\text{exp}\left(i\pi 2I_{z}S_{z}\right)$	−1	*r*

The originally proposed simple BIRD filter, nowadays called the BIRD^*d*,*X*^ filter, thus facilitates a π rotation around 2*I_y_S_y_* with phase factor 1 when the delays are matched to *J* and 2*I_z_S_x_* with phase factor −*i* for *J* = 0. The JC-BIRD, as the only case deviating from Table 1 in this article, facilitates 2*I_x_S_y_* with phase factor −1 under the matching condition and 2*I_z_S_x_* with phase factor −*i* for *J* = 0. The corresponding fidelities of spin inversion according to $\Phi_{\text{PP}}$ and propagator synthesis according to $\Phi_{\text{UR}}$ are given in Fig. 1 (C and D). For BIRD, a negative cosine dependence with *J* is observed for both cost functions, leaving a very narrow range of couplings for satisfying inversion and bilinear rotation conditions.

As far as spin inversion is concerned, the JC-BIRD pulse sequence provides improved robustness toward a variation in *J* couplings (dashed line in Fig. 1C), but in terms of the full bilinear rotation propagator, it has the same profile as the simple BIRD. It can therefore not be considered a fully *J*-compensated bilinear rotation. An overall robust bilinear rotation operation is thus highly desirable, but both hard-pulse BIRD elements also suffer from a quite narrow offset range, which shall be addressed first.

### Broadband BIRD variants

Hard-pulse BIRD elements suffer from bandwidth and *B*_1_ limitations, which are strongest for 180° pulses. Typical inversion bandwidths are smaller than the applied rf amplitude, leaving carbon as the heteronucleus *X* with a substantial problem. The application of broadband shaped pulses to overcome the problem, in principle, is straightforward, but special care has to be taken with respect to coupling evolution during shaped pulses (*32*, *41*–*43*). Therefore, previously applied adiabatic pulses or pulse sandwiches with *J* compensation are the choices for enhancing robustness.

We look at adiabatic pulses first, which qualify as inversion pulses with a defined frequency sweep. CHIRP- or Wideband, Uniform Rate, Smooth Truncation (WURST)–type shapes with linear frequency sweeps allow low-level heteronuclear *J* compensation of practical interest in ^13^C-correlated NMR due to a linear relation of chemical shifts and *J*-coupling constants in sp^2^- and sp^3^-hybridized moieties (*44*). For BIRD*^r^* and BIRD*^d^* elements, two identical adiabatic pulses with optimal timing may be applied, leading to the desired approximately linear relation with respect to $\Phi_{\text{PP}}$ and $\Phi_{\text{UR}}^{*}$ . In these cases, the two adiabatic inversion pulses add up to a 360° universal rotation. BIRD^*r*,*X*^ and BIRD^*d*,*X*^ involve only a single adiabatic pulse, which leads to the same linear relation for $\Phi_{\text{PP}}$ but cannot refocus carbon magnetization, leaving the sequence with unacceptable universal bilinear rotation performance (for a detailed simulation of the effect, see fig. S2E). The same problem persists for two adiabatic pulses applied with opposite sweep directions, previously proposed to avoid the linear *v*_S_, *J* dependence (*44*), as adiabatic pulses with opposite sweep directions generally do not compensate to a universal rotation (see fig. S2F).

**Fig. 2.**
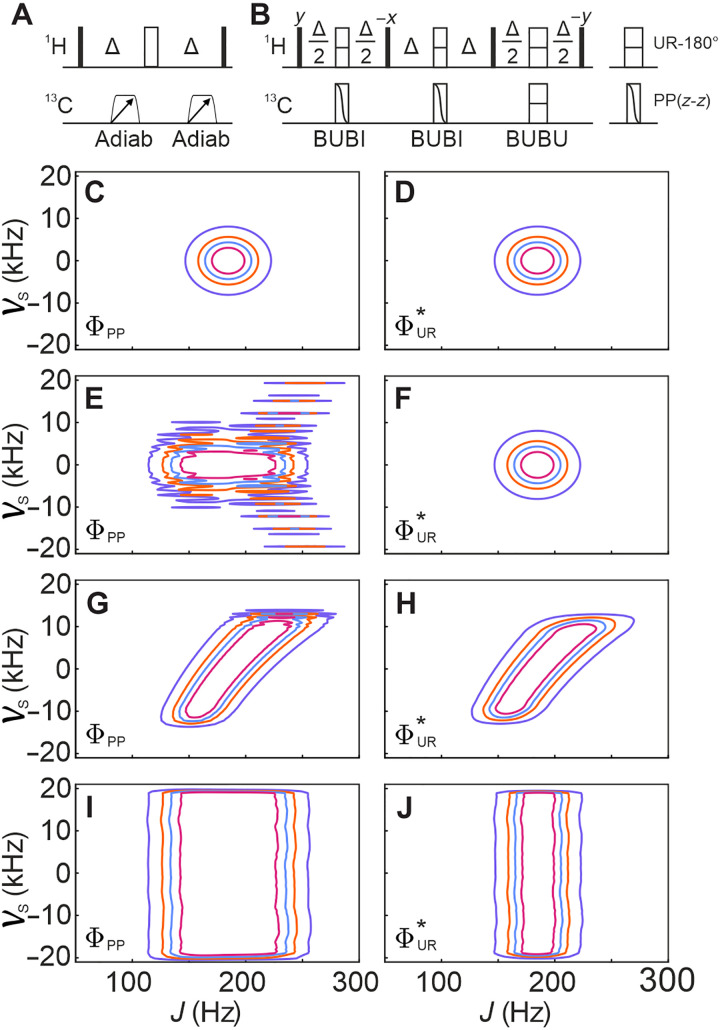
The use of offset-compensated adiabatic or optimal control–derived pulses in the BIRD and JC-BIRD to extend the robustness into offset variation. (**A**) Alternative pulse sequence for BIRD elements based on a pair of adiabatic pulses to achieve offset-compensated optimal transfer of the BIRD*^d^* bilinear rotation. Trapezoids correspond to adiabatic pulses with their sweep direction indicated by the arrows. Universal rotation refocusing (UR-180°) and point-to-point inversion pulses [PP(*z*-*z*)] are presented as open rectangles with a dash or a curved diagonal, respectively. (**B**) A JC-BIRD sequence using a pair of BUBI and a single BUBU pulse sandwich for offset compensation is shown. Transfer delays are calibrated to match Δ = 1/(2 × ^1^*J*_CH_). (**C**, **E**, **G**, and **I**) The simulations of $\Phi_{\text{PP}}$ (left column) and (**D**, **F**, **H**, and **J**) $\Phi_{\text{UR}}^{*}$ (right column) of the BIRD elements with respect to the offset frequency *v*_S_ and *J* coupling for hard-pulse BIRD*^d^* [(C) and (D)] and JC-BIRD*^d^* [(E) and (F)], as well as the offset-compensated adiabatic BIRD*^d^* [(G) and (H)] and BUBI/BUBU-JC-BIRD*^d^* [(J) and (K)]. Hard pulses were simulated with an rf amplitude of 25 kHz, corresponding to a 10-μs 90° pulse. Adiabatic WURST_40_ pulses with *T* = 1 ms, *Q* = 5, and a sweep-width Δ*v*_S_ = 40 kHz and BUBI/BUBU pulse sandwiches as specified in Table 2 were used in the simulations. Contour levels in all cases equal 0.8 (indigo), 0.9 (orange), 0.94 (ultramarine), and 0.97 (magenta).

A second solution for offset-compensated BIRD elements is based on *J*-compensated pulse sandwiches. For ^1^H,^13^C bilinear rotations, the well-established BUBI pulse sandwich, with a highly compensated proton refocusing and carbon inversion pulse (*41*), or the BUBU pulse sandwich, with *J*-compensated refocusing properties on both nuclei (*42*), may be applied. BUBI sandwiches impose substantially lower rf energy and should be the preferred choice whenever applicable, but their ^13^C inversion properties limit their application to an even number for bilinear rotations. Whenever an odd number of ^13^C pulses is required, the BUBU pulse sandwich needs to be applied at least once. Resulting pulse sequence implementations for BUBU-BIRD and BUBI-BIRD together with *v*_S_, *J* plots of $\Phi_{\text{PP}}$ and $\Phi_{\text{UR}}^{*}$ are given in the Supplementary Materials. In Fig. 2, the adiabatic BIRD*^d^* implementation with two WURST_40_ shaped pulses and the JC-BIRD^*d*,*X*^ with one BUBU and two BUBI pulse sandwiches are shown as examples, together with the simulated hard-pulse and shaped-pulse *v*_S_, *J* plots of $\Phi_{\text{PP}}$ and $\Phi_{\text{UR}}^{*}$ . The improvements of both adiabatic and optimal control theory–derived shaped pulses can be seen in the plots. However, while offsets can be nicely compensated, true *J* compensation is still only achieved for inversion with the JC-BIRD^*d*,*X*^ sequence, and more substantial changes are needed for a desired robust BIRD element.

### Optimal control–derived coupling-compensated BIRD sequences

**Fig. 3.**
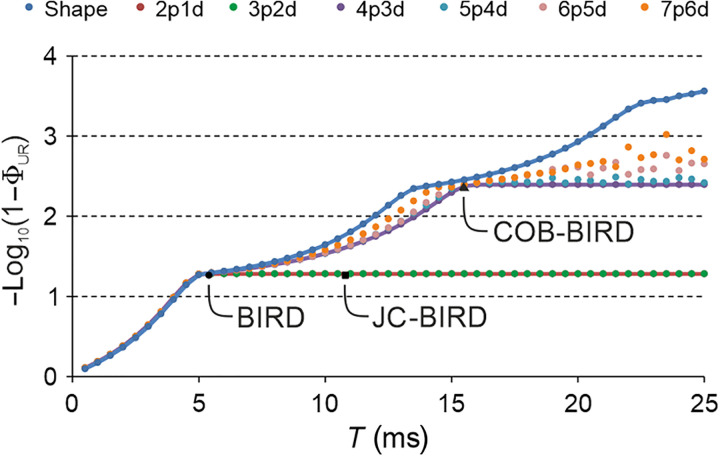
TOP performance curves for optimal control–derived JC-BIRD elements. Continuous shapes as well as hard-pulse delay sequences with, neglecting 180° pulses in refocused delays, (*n* + 1) proton pulses and (*n*) delays were evaluated using $\Phi_{\text{UR}}$ and taking only the on-resonance performance with respect to *J* into account. Different TOP curves are represented with different colors as specified on the top of the graph. While the shape TOP curve marks roughly the physical limit for the bilinear rotation, hard-pulse delay sequences are needed for actual robust implementations. The performances of the original BIRD (2p1d due to the two 90° pulses flanking the refocused delay) and JC-BIRD (4p3d) sequences as well as the 4p3d sequence used subsequently for the COB-BIRD implementation are annotated correspondingly.

From an optimization point of view, BIRD filters are *J*-selective universal rotation 180° elements that need to maximize $\Phi_{\text{UR}}$ with $U_{T}=U_{H^{d}}$ for a range of heteronuclear couplings as well as a contribution to $\Phi_{\text{UR}}$ from $U_{T}=U_{H^{r}}$ for *J* = 0. This can be plugged into an algorithm (*45*) to carry out the optimization procedure introduced in (*38*). In a first step, only the *J* dependence of the bilinear rotation is optimized, considering only the on-resonance case for both spins and ideal pulse performance. As the desired range for $U_{T}=U_{H^{d}}$ , we chose the range of 120 to 250 Hz, representing the most common range of ^1^*J*_CH_ couplings of sp-, sp^2^-, and sp^3^-hybridized carbons (*46*). Continuous pulse shapes for *I* and *S* spins were optimized with a wide range of starting conditions, resulting in a time-optimal (TOP) curve (*47*, *48*) that defines the physical limit for the shortest possible BIRD element to perform with a given overall $\Phi_{\text{UR}}$ . The logarithmic representation of the TOP curve (Fig. 3) shows several distinct plateau-like regions, where BIRD and JC-BIRD sequences only reach the first performance plateau at $\Phi_{\text{UR}}$ ≈ 0.945 starting at τ ≈ 5 ms overall delay time. After a continuous rise in performance, a second plateau is reached at τ ≈ 13 ms and a third one at τ ≈ 22 ms.

While the TOP curve is important to judge the physical limit, an actual implementation of a bilinear rotation needs to consider robustness against offset and *B*_1_ inhomogeneity. This can be introduced in a modular way in hard-pulse delay sequences by replacing hard pulses with correspondingly optimized shapes and refocusing magnetization evolving during delays (*38*, *39*). Limiting ourselves to hard-pulse delay sequences, TOP curves for (*n* + 1) pulses and (*n*) delays were calculated, labeled with (*n* + 1)p(*n*)d (Fig. 3). As a first result, we can state that 2p1d and 3p2d sequences, including the original BIRD sequence as 2p1d, generally cannot perform better than the first plateau. The second plateau requires a minimum complexity of 4p3d sequences. More complex hard-pulse delay sequences lead to even better performances, but with our approach, we could not reach the third plateau with sequences up to 7p6d and element times up to τ = 25 ms.

**Fig. 4.**
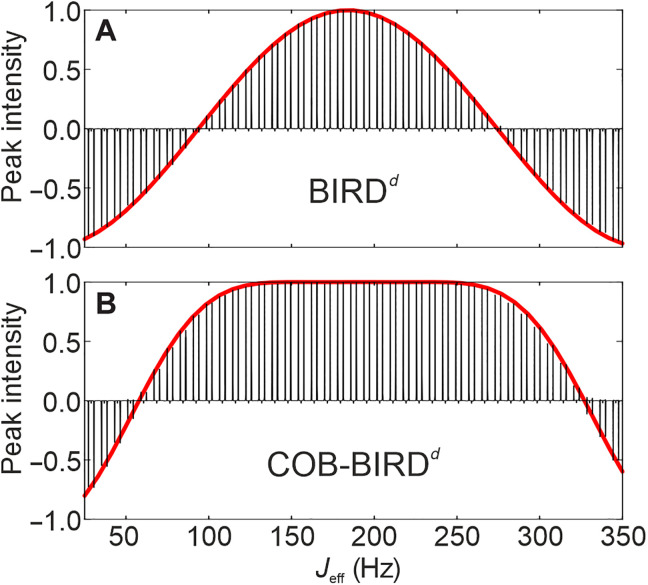
Comparison of experimental and simulated data to the universal π rotation of the BIRD*^d^* and the COB-BIRD*^d^*. Experimental demonstration (black spectral series) of the BIRD*^d^* (**A**) and the COB-BIRD*^d^* (**B**) sequences, as well as their comparison to corresponding simulations (superimposed red lines). Experimental data were obtained as described in the main text, and simulations show $\Phi_{\text{PP}}\left(I_{x}\to I^{-}\right)$ fidelities on the highlighted elements using on-resonant hard pulses. The COB-BIRD*^d^* pulse sequence is given in Fig. 5.

**Fig. 5.**
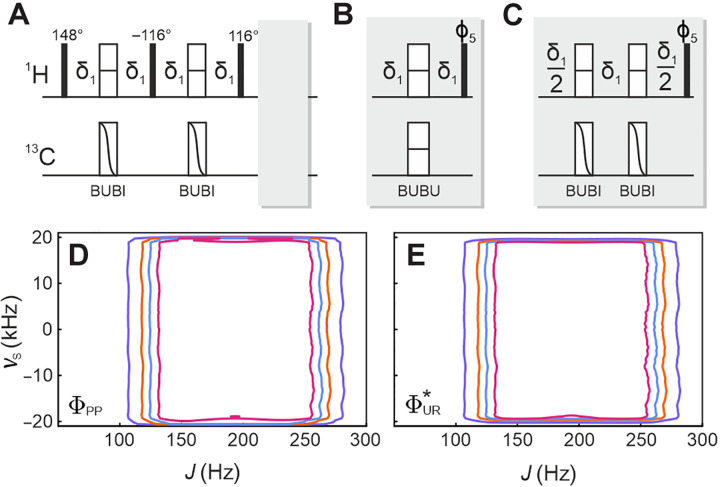
The four essential COB-BIRD pulse sequences and their experimental fidelities for spin inversion and universal π rotation against coupling and offset variation. (**A**) The general sequence for the COB-BIRD. For effective bilinear π rotations in the coupling range of 120 to 250 Hz, all delays δ_1_ = 2.583 ms. Universal rotation refocusing (UR-180°) and point-to-point inversion pulses [PP(*z*-*z*)] of pulse sandwiches are applied as indicated. By replacing the gray box with either (**B**) or (**C**) and setting the flip angle ϕ_5_ accordingly, all four different COB-BIRD bilinear rotations can be implemented. In order to design the COB-BIRD^*r*,*X*^ or the COB-BIRD^*d*,*X*^, fragment (B) is used with ϕ_5_ set to −148° or 32°, respectively, and in order to design the COB-BIRD*^d^* or COB-BIRD*^r^*, fragment (C) is used with ϕ_5_ set to −148° or 32°, respectively. As an example, the $\Phi_{\text{PP}}$ (**D**) and $\Phi_{\text{UR}}^{*}$ (**E**) fidelities for the COB-BIRD^*r*,*X*^ variant are simulated with respect to *v*_S_ and *J*. Contours are scaled the same way as in Fig. 2.

Since a BIRD element in an actual experiment will have to take into account relaxation and added time from real pulses, we chose a 4p3d and a 5p4d sequence out of the multitude of TOP hard-pulse delay sequences for further refinement. Both have a duration of 15.5 ms and performance $\Phi_{\text{UR}}$ ≈ 0.996. In addition, the 4p3d obeys the symmetry principle derived to obtain universal rotation pulses (*49*). We simulated their performance and implemented them experimentally (Fig. 4) using a 140 mM sodium acetate-2-^13^C sample (^1^*J*_CH_ = 125.3 Hz) dissolved in a 1:5 (v/v) mixture of D_2_O/dimethyl sulfoxide (DMSO)–*d*_6_ for a well-defined spin system emulating effective *J*-coupling dependencies by scaled delays, as described in the Supplementary Materials. In all cases, the correspondence between theory and experiment is good. However, for brevity, we limit the description here to the 4p3d sequence (Fig. 5), while corresponding data for the 5p4d sequence can be found in the Supplementary Materials.

### Fully compensated COB-BIRD variants

So far, coupling-compensated BIRD sequences have been derived with hard pulses with poor *B*_1_ and offset-related properties. As described in the COB recipe (*38*) for coupling, offset, and *B*_1_ compensation, the hard pulses in a second step have to be replaced by correspondingly compensated shaped pulses. In the following, we consider the needs of a ^1^H,^13^C COB-BIRD element applied on a 600-MHz spectrometer, for which a typical ^1^H bandwidth of 16.7 parts per million (ppm) and a ^13^C bandwidth of 250 ppm (*50*) correspond to 10 and 37.5 kHz, respectively. For variation in *B*_1_, a range of ϑ = ±20% of the nominal rf amplitude is assumed for ^1^H and ϑ = ±5% for ^13^C pulses. Pulse shapes have been optimized for the 5p4d and 4p3d sequences, as well as for broadband versions of the original BIRD and JC-BIRD. For the experimental verification, we also used the COB3-INEPT (*39*) element with specifically optimized shaped pulses to transform antiphase signal into in-phase for ^1^H-decoupled detection on ^13^C. The COB3-INEPT element has been optimized for having a *J*-compensated INEPT transfer for couplings in the range of 120 to 750 Hz. In our application, it guarantees highly efficient transfer from ^1^H,^13^C antiphase to ^13^C in-phase magnetization for CH and CH_2_ groups and fair transfer for CH_3_ groups (*39*). We used the already published BUBI and BUBU pulse sandwiches and optimized all other universal rotation pulses using self-written code (*40*, *42*, *51*–*54*). All pulse shapes that are necessary for all offset-compensated sequences introduced in Figs. 2 and 5 and the COB3-INEPT are summarized in Table 2.

**Table 2.** All parameters and the corresponding fidelity of the shaped pulses used in the main text. For each shape, in order, the pulse time (*t*_p_), total number of digits (# dig), the controlled bandwidth (BW), maximum rf amplitude (RF), *B*_1_ compensation around the nominal rf amplitude ( ϑ ), and the corresponding fidelity are presented. Pulse types refer to xyBEBOP saturation pulses (*66*–*69*) , BEBOP (*70*, *71*) and BIBOP (*47*) point-to-point excitation and inversion pulses, BURBOP universal rotation pulses (*40*, *72*), and the ^1^H and ^13^C parts of the BUBI (*41*) and BUBU (*42*) pulse sandwiches.

Type	*t*_p_ (μs)	# dig	BW (kHz)	RF (kHz)	ϑ (%)	Fidelity
WURST_40_	1000	1000	20	5.6	0	0.99898
WURST_40_	2000	1000	25	4.0	0	0.99920
xyBEBOP	300	300	37.5	10	5	0.99999
BEBOP (*z* → −*y*)	150	150	10	20	20	0.99994
BEBOP (*z* → −*y*)	700	700	37.5	10	5	0.99965
BIBOP (*z* → −*z*)	200	200	10	20	20	0.99997
BURBOP 32*_x_*	200	200	10	20	20	0.99998
BURBOP 90*_x_*	200	200	10	20	20	0.99997
BURBOP 90*_x_*	700	700	37.5	10	5	0.99946
BURBOP 116*_x_*	200	200	10	20	20	0.99997
BURBOP 136*_x_*	800	800	37.5	10	5	0.99970
BURBOP 148*_x_*	200	200	10	20	20	0.99995
BURBOP 180*_x_*	1000	1000	37.5	10	5	0.99990
BUBI(^1^H)	600	1200	10	20	20	0.99998
BUBI(^13^C)	600	1200	37.5	10	5	0.99914
BUBU(^1^H)	1000	2000	10	18.5	20	0.99998
BUBU(^13^C)	1000	2000	37.5	20	5	0.99991

The performance of the fully compensated COB-BIRD sequence can be seen in the simulation for the offset dependence in Fig. 5. Both $\Phi_{\text{PP}}$ and $\Phi_{\text{UR}}$ demonstrate the exceptional behavior of COB-BIRD for a truly *J*-compensated bilinear rotation element for the full range of couplings and optimized offsets. When all hard pulses in the COB-BIRD sequence are replaced with their shaped counterparts, also the overall performance of the filter element is robust. Using the toolkit presented in Fig. 5 (A to C), all effective bilinear rotations can be reproduced as presented in Table 1: A single BUBU pulse sandwich during the last COB-BIRD delay will lead to inversion of the *X* nucleus, while it is unchanged if the last COB-BIRD delay is applied with a pair of BUBI sandwiches. Correspondingly, setting the flip angle to ϕ_5_ = −148°, a COB-BIRD^*r*,*X*^ or COB-BIRD*^d^*, respectively, is obtained, while setting to ϕ_5_ = 180° − 148° = 32° results in a COB-BIRD^*d*,*X*^ or COB-BIRD*^r^* element. Only the performance of the COB-BIRD^*r*,*X*^ is shown in Fig. 5, but all other versions of the COB-BIRD perform equally well (also see the Supplementary Materials).

### Experimental comparison

For an experimental demonstration of the performance of the different BIRD elements, we designed a ^13^C-detected pulse sequence for the measurement of the total coupling *T* = *J* + 2*D* in partially aligned samples. As an example case, we looked at 100 mM (−)-nicotine dissolved in a lyotropic 9% poly-γ-benzyl-l-glutamate (PBLG)/CDCl_3_ mesophase with total heteronuclear one-bond couplings ranging from 71 to 253 Hz. In the viscous solution, ^1^H and ^13^C *T*_2_ relaxation times on the order of few hundreds of milliseconds are obtained (see table S4).

**Fig. 6.**
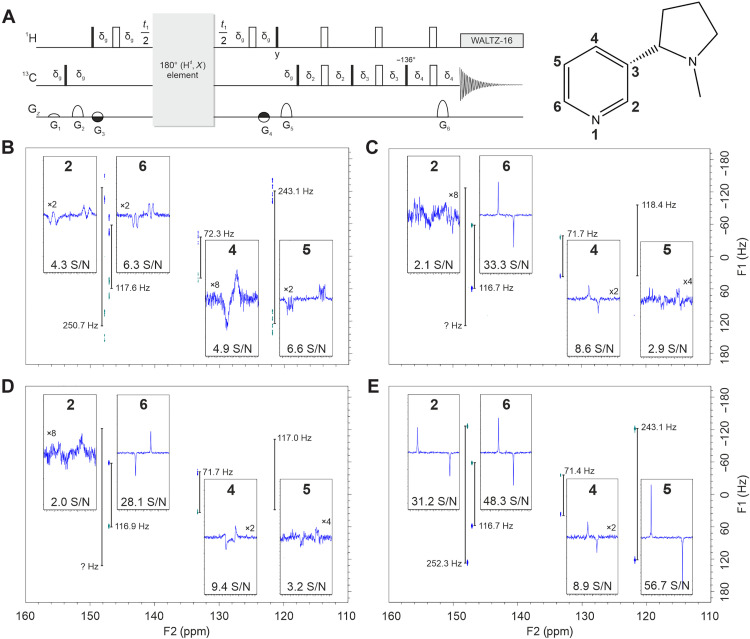
Experimental comparison of various refocusing elements applied in a ^13^C-detected, ^1^H-decoupled, and refocused *J*-INEPT experiment. (**A**) The corresponding sequence with a gray box for different inversion elements and a COB3-INEPT transfer step is shown together with the structure and aromatic numbering of nicotine on top of the spectra. All pulses are applied along *x* unless indicated otherwise, and solid and open bars refer to 90° and 180° pulses, respectively, unless a different flip angle (136°) is annotated. For the refocusing elements, a simple 180° hard-pulse spin echo (**B**) is compared to the BIRD^*d*,*X*^ (**C**), the JC-BIRD^*d*,*X*^ (**D**), and the COB-BIRD^*d*,*X*^ (**E**) elements. All four 2D spectra were applied to a partially aligned sample of (−)-nicotine as explained in the main text. For each experiment, slices of the aromatic signals are extracted over the F1 dimension and shown with the corresponding measured *T* coupling and signal-to-noise-ratio (S/N). The transfer delays in the BIRD^*d*,*X*^ and JC-BIRD^*d*,*X*^ are set to 125 Hz to accommodate the small couplings, and the COB-BIRD^*d*,*X*^ is applied as presented in Fig. 5. Gradients of 1-ms duration and 200-μs recovery delay are applied in percent of the maximum gradient strength of nominally 53.5 G/cm according to G_1_ = 7%, G_2_ = 25%, G_3_ = −G_4_ = 10%, G_5_ = 60%, and G_6_ = 80%. Delays are δ_g_ = 1.2 ms, δ_2_ = 0.5401 ms, δ_3_ = 1.065 ms, and δ_4_ = 1.0702 ms. For a detailed description including phase cycling and the particular 180° element used, refer to the Supplementary Materials (fig. S14).

The experiment applied is shown in Fig. 6. It is a refocused *J*-INEPT experiment with a *J*-evolution period for the indirect dimension after starting from ^1^H magnetization and a transfer step for refocusing antiphase into heteronuclear decoupled in-phase ^13^C magnetization during acquisition. In the center of the *t*_1_ period, an inversion element is implemented for effective *J* evolution, which can be either a simple 180° pulse or any BIRD^*d*,*X*^ element, where the latter ones should result in decoupling of all homonuclear couplings to remote protons. For the INEPT transfer, the highly compensated COB3-INEPT (*39*) is used, but with the nuclei inverted compared to the originally published sequence, making it necessary to optimize several pulse shapes specifically for this purpose (see Table 2). Four different versions of the experiment were implemented and applied to the partially aligned sample, using a 180° pulse, a BIRD^*d*,*X*^, a JC-BIRD^*d*,*X*^, and a COB-BIRD^*d*,*X*^ in the center of *t*_1_. Resulting spectra are shown in Fig. 6.

In all cases, we focused on the aromatic region of (−)-nicotine, in which most extreme total couplings are observed in CH groups. Because of a multitude of ^1^H,^1^H couplings, ^1^*J*_CH_ coupling measurement is rather inaccurate in the spectrum without filter element (Fig. 6A). In the other spectra, the signal at $\delta \left({}^{13}C\right)$ = 148 ppm with a coupling constant of 117 Hz shows good decoupling performance and good sensitivity with all BIRD^*d*,*X*^ sequences. Couplings can still be measured with acceptable accuracy for the signal at $\delta \left({}^{13}C\right)$ = 133 ppm to 71 to 72 Hz, although sensitivity is highly reduced because of the mismatched coupling for all BIRD elements and the COB3-INEPT transfer step. Besides the low sensitivity, the *J*-coupling mismatch also results in a phase-twisted signal for the JC-BIRD^*d*,*X*^ [signal-to-noise-ratio (S/N) is measured after phasing the one-dimensional (1D) slice separately; otherwise, signal intensity would be substantially lower]. The signals at $\delta \left({}^{13}C\right)$ = 148 ppm and $\delta \left({}^{13}C\right)$ = 122 ppm have couplings of 252 and 243 Hz, respectively. Spectra for BIRD^*d*,*X*^ and JC-BIRD^*d*,*X*^ show unphaseable, low-intensity signals for these large couplings, indicating that, expectedly, Φ_UR_ is the adequate fidelity function for the shown application where all BIRD filters are used as refocusing elements in the center of *t*_1_ evolution. The COB-BIRD^*d*,*X*^ element, instead, shows outstanding performance with respect to decoupling and S/N for the large couplings as expected from simulations.

Equivalent results for refocused INEPT-type spectra using BIRD*^r^* variants for homonuclear decoupling in the indirect dimension are shown in the Supplementary Materials. Resulting decoupling and sensitivity performances are essentially equivalent to the *J*-resolved case. For an example of a slightly larger molecule in isotropic solution, we recorded the COB-BIRD^*d*,*X*^ *J*-INEPT experiment also on an 11-mer peptide, with the resulting spectra and relaxation data summarized in the Supplementary Materials.

## DISCUSSION

### Experimental considerations

The different classical BIRD elements as bilinear π rotations form the basis of a vast multitude of NMR applications. They treat protons with a heteronuclear coupling close to *J* = 0 different from protons that are coupled to the same heteronucleus around a nominal coupling *J*^nom^. However, hard pulses lead to poor performance with respect to offset and *B*_1_ inhomogeneity, and several improvements in terms of robustness have been derived here.

As a first improvement, broadbandedness has been addressed by shaped pulses for the heteronucleus ^13^C. The first lesson learned is that the BIRD elements behave quite differently in different types of applications. The most easily fulfilled condition is required in experiments that only use the inversion properties of BIRD elements. Examples are so-called *X*-filtered experiments for isotope selection or spectral cleanup in heteronuclear correlation experiments to a low natural abundance nucleus without gradients. In such cases, magnetization is best stored along *z* before the BIRD element, and the point-to-point quality factor $\Phi_{\text{PP}}\left(z\to -z\right)$ is sufficient to describe corresponding properties.

The situation changes substantially if magnetization is in the transverse plane before the BIRD element, as is the case in all experiments designed for direct/remote selective decoupling in an indirect dimension. Whenever a BIRD element is placed in a *t*_1_ period, *x*,*y* magnetization on either the *I* or the *S* spin needs to be refocused, implying a more difficult to achieve universal rotation quality factor like $\Phi_{\text{UR}}^{*}$ . In particular, Heteronuclear Single Quatum Coherence (HSQC)–type experiments with transverse ^13^C magnetization during *t*_1_ require $\Phi_{\text{UR}}^{*}$ . Other experiments, in which only ^1^H magnetization is transverse during the *t*_1_ period, are either described by $\Phi_{\text{UR}}^{*}$ or the more specific ΦUR(H1)* , which is discussed in detail in the Supplementary Materials.

In general, the best results are obtained by *J*-compensated BUBI and BUBU pulse sandwiches (*41*, *42*) that allow the implementation of uniform bilinear rotations at ≈*J*^nom^. Adiabatic pulses, instead, allow full offset-compensated bilinear rotations only for BIRD*^d^* and BIRD*^r^* elements with the application of two identical adiabatic pulses on the heteronucleus and the sometimes beneficial effect of a linear correlation of *v*_S_ and *J*. Other introduced sequences with adiabatic pulses may still be applicable if only ^1^H π rotations are needed and simple inversions are sufficient for ^13^C. Specific examples, where the adiabatic approach fails, concern ^13^C *J*-resolved spectra with a BIRD^*d*,*X*^ filter for selective decoupling like in (*55*, *56*). A detailed collection of the different BIRD elements described in this article with respect to different quality factors is given in the Supplementary Materials.

A second type of improvement concerns the extension of the BIRD elements toward a range of nominal couplings $J^{\text{nom}}\in \left[120\;\text{Hz},250\;\text{Hz}\right]$ . Such a range of couplings is needed for applications concerning all carbon hybridizations, or when residual dipolar couplings extend the conventional range of couplings. The JC-BIRD element, already introduced in the original BIRD publication (*4*), allows *J*-compensated bilinear inversions on ^1^H, which is sufficient for simple applications such as *X* filtering. However, full bilinear rotations are only obtained by the currently introduced COB-BIRD sequences. Together with specifically designed broadband universal rotation pulses, robust compensation for ranges of couplings, offsets, and *B*_1_ values is obtained. Optimized TOP curves make certain that the sequences are close to the physical limit.

Which of the bilinear rotation experiments should be used in a specific application depends on the effective distributions of couplings and offset ranges of specific samples, as well as the *B*_1_ distribution of a particular probehead used. In addition, relaxation properties play a role especially for large molecules with short *T*_2_ times, for which the 15.5-ms delay time of the COB-BIRD sequences may lead to intolerable results. However, for small-molecule applications with a single BIRD-type element, experiments shown here demonstrate the impressive benefit from the COB-BIRD.

Applications with other heteronuclei such as ^15^N or in other fields of quantum technologies will require other ranges of couplings or will have other needs in terms of offset/detuning, etc. In particular, gate-based quantum technology needs the full bilinear rotation properties of the sequence elements. Pulses may easily be reoptimized for specific needs in such cases using standard implementations of the GRadient Ascent Pulse Engineering (GRAPE) algorithm as provided by Spinach (*57*–*60*), Simpson (*61*, *62*), Seedless (*63*), or Octopussi (*41*, *47*, *52*, *64*, *65*), to name only a few programs. Specific deviating ranges of nominal *J* couplings may be obtained by linearly scaling the delays in COB-BIRD sequences. Making the sequence delays twice as long, for example, will result in a nominal range of $J^{\text{nom}}\in \left[60\;\text{Hz},125\;\text{Hz}\right]$ . If specific needs deviate substantially from scaled ranges, the approach presented here at least provides the viable optimization scheme as such for an efficient adapted optimization.

The application of all BIRD sequences studied here is straightforward for nonchiral molecules. In chiral molecules, CH_2_ groups with large ^2^*J*_HH_ couplings may result in phase distortions due to homonuclear geminal coupling evolution during BIRD. The effect does not directly scale with the length of the BIRD element but should be strongest for JC-BIRD and COB-BIRD. More substantial distortions are expected for coupling networks in the strong coupling limit with second-order artifacts, independent of the type of element. Strong coupling artifacts have been reported to being reduced by the application of an *XY* − 4 supercycle (*4*), which can reduce some of the artifacts but will also lead to the application of four BIRD elements in a single experiment, which will increase the effect of relaxation on the acquired spectra.

The extension of the COB-optimization approach to other specific bilinear rotations, such as TANGO (*28*), should be straightforward. For general bilinear rotation concepts with variable rotation angles (*25*–*27*), it is currently unclear whether the results may be transferred to a range of couplings. However, for individual rotation angles, the approach should be applicable.

### Final remarks

COB-BIRD elements have been introduced after a systematic study of previously reported BIRD filters, which have also been improved in terms of robustness by adiabatic pulses and optimal control–derived pulse sandwiches, and a general search for the physical limits for *J* compensation in BIRD-type elements has been performed. The resulting sequences allow specific bilinear π rotations distinguishing remote protons with heteronuclear *J* couplings close to zero from direct protons with corresponding couplings in the range of 120 to 250 Hz. The presented COB-BIRD sequences are designed for application in typical high-resolution 600-MHz spectrometers, but they also provide a general recipe for other practical setups, as, in this case, only a small number of universal rotation pulses need to be reoptimized using, for example, standard implementations of the GRAPE algorithm (*41*, *47*, *52*, *57*–*59*, *61*–*65*).

We expect that the different COB-BIRD variants will have many applications in NMR spectroscopy, particularly as decoupling elements in dimensions with time incrementation. It also represents a viable role model for the design of robust pulsed gates in various areas of quantum technology applications.

## MATERIALS AND METHODS

All *J*-coupling profile experiments as shown in Fig. 2 and fig. S8 were obtained as a series of 1D experiments on a 500-MHz Bruker Avance III HD spectrometer equipped with a CryoProbe Prodigy. The spectra were recorded at 300 K on a 140 mM sample of sodium acetate-2-^13^C in 1:5 (v/v) D_2_O/DMSO-*d*_6_ with a ^1^*J*_CH_ measured to 125.3 Hz. All spectra were recorded with 1.5-kHz bandwidth irradiated at 1.65 ppm. To avoid the effects of *B*_1_ inhomogeneities and *J* couplings during concurrent 180° pulses, we used BUBI shaped pulse sandwiches. Hard pulses were used for the odd-flip angle rotations. All shaped ^1^H and ^13^C pulses have been calibrated to a maximum rf amplitude of 20 and 10 kHz, corresponding to 12.5- and 25-μs 90° pulse lengths, respectively. Data were collected for 8192 complex data points and zero-filled to 16,384 complex data points. No apodization was performed, and after the Fourier transform, the frequency data were phased and subjected to an automated baseline correction.

All 2D INEPT–type experiments on the partially aligned (−)-nicotine sample were performed on a 600-MHz Bruker Avance III spectrometer equipped with a cryogenically cooled inverse triple resonance probehead (^1^H, ^13^C, and ^15^N) with *z* gradient (Bruker BioSpin GmbH, Rheinstetten, Germany) using a 100 mM (−)-nicotine dissolved in a lyotropic 9% PBLG/CDCl_3_ mesophase measured at 298 K. The sample was mixed inside the NMR tube by dissolving 8 μl of (−)-nicotine in 500 μl of CDCl_3_, after which, 74.5 mg of PBLG was added. The PBLG was effectively dissolved using repetitive short centrifuging of the NMR tube. The resulting solvent CDCl_3_ of the lyotropic mesophase in the 600-MHz spectrometer was measured to have a quadrupolar coupling Δ*v*_Q_ of 331.4 Hz.

All spectra recorded on the spectral range of the aromatic ^13^C and ^1^H frequencies, as presented in Fig. 6 and fig. S9, are recorded using the pulse program as presented in fig. S14 using BIRD^*d*,*X*^ variants during *t*_1_ evolution. The spectra were recorded with 16 dummy scans, 16 scans per increment, and 1.0-s relaxation delay over spectral ranges of 7.5 kHz by 1.8 kHz irradiated at 135 and 6 ppm for ^13^C and ^1^H, respectively, using hard pulses. Data were recorded for 8192 × 1024 complex data points and zero-filled to 16,384 × 2048. For all experiments involving a BIRD or JC-BIRD element, the delays were set to match a ^1^*T*_CH_ coupling of 125 Hz. The time-domain data were apodized with a 90°-shifted quadratic sine function over both dimensions for coupling measurement, while for display purposes in Fig. 6 and figs. S9 and 10, this apodization was replaced by a 10-Hz exponential decay. Effective proton decoupling during acquisition was achieved using the WALTZ-16 supercycle. After performing the Fourier transform, a manual phase correction was executed, and the frequency data were subjected to an automatic baseline correction.

For the 2D experiments over the full range of frequencies as presented in fig. S10, spectra were recorded with shaped pulses as presented in table S3. The spectra were recorded with 16 dummy scans, 16 scans per increment, and 2.0-s relaxation delay over spectral ranges of 21 kHz by 4.7 kHz irradiated at 87 and 3.5 ppm for ^13^C and ^1^H, respectively. Data were recorded for 16,384 × 1024 complex data points and zero-filled to 32,768 × 2048. The time-domain data were apodized with a 90°-shifted quadratic sine function over both dimensions for coupling measurements, while for display purposes, a 10-Hz exponential decay function was used instead in the direct dimension. The COB-BIRD^*d*,*X*^ experiment on the 11-nucleotide oligomer peptide was performed on a 850-MHz Bruker Avance III spectrometer equipped with a cryogenically cooled inverse triple resonance probehead (^1^H, ^13^C, and ^15^N) with *z* gradient (Bruker BioSpin GmbH, Rheinstetten, Germany) using a sample prepared by dissolving 30 mg of the peptide in 600 μl of D_2_O. Data were recorded for 16,384 × 512 complex data points with 16 scans and zero-filled to 16,384 × 1024 with a ^13^C spectral window of 55 ppm and 400 Hz over F2 and F1, respectively, and with transmitter frequencies at 35 and 3 ppm for ^13^C and ^1^H, respectively. The time domain data were apodized with a 90°-shifted quadratic sine function over both dimensions. (−)-Nicotine was obtained from Carl Roth at ≥99% purity. PBLG (average molar mass of 150,000 to 350,000 g/mol) was obtained from Sigma-Aldrich. CDCl_3_ (H_2_O of <0.01%), DMSO-*d*_6_, and D_2_O were obtained from Eurisotop, and CDCl_3_ was stabilized over silver foil. Sodium acetate-2-^13^C was obtained from Sigma-Aldrich. All chemicals were used without further purification.

### Supplementary Material

Detailed results for the original BIRD sequence; details on adiabatic pulses used; all pulse sequences discussed in the text; the three different quality factors $\Phi_{\text{PP}}$ , $\Phi_{\text{UR}}^{*}$ , and the additionally derived ΦUR(H1)* plotted with respect to *v*_S_ and *J*; a summary of all pulse shapes used; details of the optimizations with top curves of shaped and hard-pulse delay sequences; details on experimental verification; and data for the *v*_S_-evolved refocused INEPT sequence equivalent to Fig. 6 are given in the Supplementary Materials.
