# Supplementary material for: Robust bilinear rotations
Source: Sci Adv. 2025 Aug 29;11(35):eadx7094. doi: 10.1126/sciadv.adx7094 (PMC12396312; doi:10.1126/sciadv.adx7094)
Supplement: Supplementary file 1 — Supplementary Text Figs. S1 to S14 Tables S1 to S5 [file sciadv.adx7094_sm.pdf]

Science Advances
AAAS

Supplementary Materials for

Robust bilinear rotations

Yannik T. Woordes *et al.*

Corresponding author: Burkhard Luy, burkhard.luy@kit.edu

Sci. Adv. **11**, eadx7094 (2025)
DOI: 10.1126/sciadv.adx7094

This PDF file includes:

Supplementary Text
Figs. S1 to S14
Tables S1 to S5

1 Effective Universal Rotations for different BIRD elements including phase factors

In Figure S1 the basic BIRD element with its four different phases ϕ_1 - ϕ_4 is shown. Depending on the phases, different effective propagators of the form

$$U_{\mathrm{H^d}} = \exp\left(-i\pi 2I^d_\alpha S_\beta\right) \quad \text{(S1)}$$

with $\alpha, \beta \in \{x,y,z\}$ are produced. However, the propagator analysis of a two-spin system hides certain subtleties, one of them being the propagator phase factor. The fact that the phase factor of a propagator does not affect the final state of the magnetization but plays a major role in pulse sequence design, as was extensively discussed in (40) for isolated single-spin systems, allowed that phase factors can be determined as follows.

Given that

$$\det(e^A) = e^{\mathrm{Tr}(A)} \quad \text{(S2)}$$

and the nuclear spin Hamiltonian is a traceless matrix, the determinant of any unitary single-spin propagator has to yield unity. The same has to hold for propagators with a phase factor $e^{i\phi}$. Following

$$\det(rA) = r^n \det(A) \quad \text{(S3)}$$

with n being the dimension of the matrix,

$$\det(e^{i\phi}U) = e^{in\phi}\det(U) = 1 \quad \text{(S4)}$$

has to be fulfilled. This is the case if

$$e^{in\phi} = \cos n\phi + i\cdot \sin n\phi = 1 \quad \text{(S5)}$$

is fulfilled. Possible solutions for ϕ for a single spin are integer multiples of π so that phase factors of ± 1 are obtained (40). For two-spin systems, n in equation (S3) equals four and the solutions for ϕ are integer multiples of $\pi/2$ so that phase factors of ± 1 as well as $\pm i$ are allowed. Applying the complex square of the trace in Φ_{UR} removes the phase factor and simplifies further analysis. For a pulse sequence optimization, instead, the detailed description of the bilinear rotations including its phase is essential. A useful relation in this context is that imaginary phase factors can be used to transform concurrent or even consecutive rotations around the axes corresponding to commuting operators I and S into a single bilinear rotation via

$$\exp(-i\pi(I+S)) = -i\cdot \exp(-i\pi 2IS). \quad \text{(S6)}$$

This relation will be a great boon in propagator analysis and optimizations. After determining Φ_{PP} for the single-spin operator transformations of both spins individually, the phase factor of the bilinear rotation can be determined from a simple guess. We applied the approach to analyse the rotation properties of the pulse sequences given in Figure S1. The originally proposed simple BIRD filter thus facilitates a rotation around $2I_yS_y$ with phase factor 1 when the delays are matched to J and S_x with phase factor -1 for J = 0. The J-compensated sequence facilitates $2I_xS_y$ with phase factor -1 under the matching condition and $2I_zS_x$ with phase factor $-i$ for J = 0.

In order to provide the necessary information for a feasibility study by OCT, the propagator analysis was extended to all BIRD variants proposed in (5) and the propagators and phase factors which can serve as input for optimizations are compiled in Table S1.

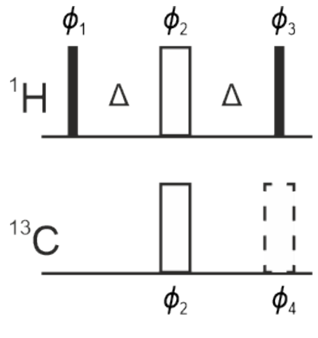

Fig. S1: **Original BIRD element and with adjustable phases for the design of the BIRD^{d,X}, BIRD^{r,X}, BIRD^{d}, and BIRD^{r}.** Pulse sequences discussed in the original BIRD publication (4) and its variants as dissused by (5). Narrow and wide bars correspond to 90° and 180° pulses, respectively. Transfer delays are calibrated to match Δ = 1/(2$^1J_{\mathrm{CH}}$). The phases ϕ_1 to ϕ_3 in the original pulse sequence (A) can be set according to (5) to achieve BIRD^{d,X} and BIRD^{r,X} manipulations. The dashed 180° pulse with phase ϕ_4 can be set to achieve the corresponding BIRD^{d} and BIRD^{r} manipulations. All possible combinations of ϕ_1 to ϕ_4 to achieve different effective bilinear rotations are presented in Table S1.

ϕ_1	ϕ_2	ϕ_3	ϕ_4	U_{H^r}	$e^{i\phi}$	U_{H^d}	$e^{i\phi}$	Descriptor
x	x	x	-	S_x	-1	$2I_yS_y$	1	d,X
x	x	x	x	1	1	$2I_yS_z$	1	d
x	x	x	y	S_z	1	$2I_yS_y + S_y$	1	d
x	x	$-x$	-	$2I_xS_x$	$-i$	$2I_zS_y$	-1	r,X
x	x	$-x$	x	I_x	-1	$2I_zS_z$	-1	r
x	x	$-x$	y	$2I_xS_z$	i	$2I_zS_y + S_y$	-1	r
x	y	x	-	$2I_yS_y$	$-i$	S_x	$-i$	r,X
x	y	x	x	$2I_yS_z$	$-i$	1	i	r
x	y	x	y	I_y	-1	S_z	i	r
x	y	$-x$	-	$2I_zS_y$	i	$2I_xS_x$	1	d,X
x	y	$-x$	x	$2I_zS_z$	i	I_x	$-i$	d
x	y	$-x$	y	I_z	1	$2I_xS_z$	-1	d

Table S1: **Rotational axis and phase factors of all known BIRD elemets.** Known BIRD rotations characterized by their descriptor as introduced in (5) as well as the rotation axes of the effective propagators acting on directly and remotely-bound protons (U_{H^d} and U_{H^r}, respectively) with their respective phase factors $e^{i\phi}$ as a function of the phases ϕ_1 to ϕ_4.

2 Calculation of adiabatic pulses used for simulations

To evaluate the ^{1}H spin inversion and bilinear rotation properties as a function of ν_S and J for adiabatically compensated carbon pulses, corresponding shapes need to be calculated properly to match the linear dependence of chemical shifts and J-couplings. The recipe for optimal pulse element lengths T and durations of transfer delays δ given in (44) to correlate a given offset range $\Delta\nu_S$ with a range of J-couplings in BIRD elements assumes

$$nT_{\text{opt}} = \frac{1}{2J_{\text{min}}} - \frac{1}{2J_{\text{max}}}. \tag{S7}$$

where n is the number of adiabatic pulses in the element, generally either 1 or 2. We chose to use J_{min} = 125 Hz and J_{max} = 250 Hz as input for equation (S7), which yields T_{opt} = 1 ms. The optimal delay δ_{opt} can be found via

$$\delta_{\text{opt}} = \frac{1}{2J_{\text{min}}} \tag{S8}$$

which equates to δ_{opt} = 4 ms and will include the time of the adiabatic pulse if present. Using the values from above a total duration of 8 ms is found for this particular BIRD element. We designed a WURST-40 pulse with T = 1 ms, Q = 5 and a sweep-width $\Delta\nu_S$ = 40 kHz for evaluations with respect to Φ_{PP}, Φ_{UR}, and Φ^*_{UR}. Given that the double-sweep layout is used for the purpose of J-compensation, a BIRD^d rotation is obtained. If a BIRD^{d,X} rotation is desired, the alternatively proposed single-sweep layout can be used (44), leading to a T = 2 ms WURST pulse with Q = 5 and $\Delta\nu_S$ = 40 kHz with two delays of 4 ms (including the pulse time). The WURST pulse and its phase-reversed version were used in the case of adiabatic sweeps with opposed directions. It can be seen that if an offset ν_S is efficiently swept by the adiabatic pulses, efficient transfer is achieved for J-couplings around a single optimal value corresponding to 167 Hz for δ = 3 ms.

3 Universal ^{1}H rotation performance of BIRD elements

In the main text, quality factors for describing the I_z inversion (Φ_{PP}) as well as the overall universal rotation (Φ^*_{UR}) performances have been introduced. However, often only ^{1}H universal rotations matter, like in the case of homonuclear decoupled spectra shown for partially align (-)-nicotine. The carbon universal rotation performance in such cases is of no relevance. For evaluating this case, another cost function may be introduced by

$$\Phi^*_{\text{UR}(^1\text{H})} = 2\,\langle U_{\text{T}}(I,J)\; U_{\text{eff}}(S) |\; U_{\text{eff}}(I,J,S)\rangle^2 - 1, \tag{S9}$$

where the propagator $U_{\text{eff}}(I,J,S)$ involves all contributions of the two-spin system, while $U_{\text{eff}}(S)$ contains only the linear contributions on the S spin, following the principle previously introduced in (41). Taking the example of BIRD^{d,X} or BIRD^{r,X} type filters, $U_{\text{T}}(I,J) = \exp(i\pi 2I_yS_z)$ or $U_{\text{T}}(I,J) = -\exp(i\pi 2I_zS_z)$, respectively, is applied in simulations. It can be noted that in all cases the change in the bilinear rotation from $U_{\text{T}}(I,J,S)$ to $U_{\text{T}}(I,J)$ essentially puts the S rotation to

S_z, which also means that for BIRD^d and BIRD^r and their adapted variants $U_T(I,J,S)$ to $U_T(I,J)$ only potentially differ in their phases, which is irrelevant in the simulation of Φ^*_{UR} and $\Phi^*_{\mathrm{UR}(^1\mathrm{H})}$. All bilinear rotations used in the simulations are presented in Table S2. The ν_S- vs. J-dependence of all cost functions for the various sequences discussed in the article are displayed in the Figure S2 and S3.

3.1 Remark regarding the appearance of different quality factors

One of the reviewers noted during revision that $\Phi^*_{\mathrm{UR}(^1\mathrm{H})}$ generally shows worse results than Φ^*_{UR} although it only describes the ^{1}H subset and should therefore be better in performance. It is also noticeable that Φ_{PP} as well as $\Phi^*_{\mathrm{UR}(^1\mathrm{H})}$ show distinct stripe-like behavior along the carbon chemical shift range ν_S whenever two or more refocused delays are present in the sequence. To understand the two observed effects, let's have a closer look at what happens in such a case.

Let's assume perfectly matched delays at a substantial offset of spin S. In this case, inphase I_x magnetization is transformed to $2I_yS_z$ during the first half of the first refocused delay. The 180° hard pulse along x will now not fully invert the magnetization to $-2I_yS_z$, but will lead to partial inversion with resulting $-a\ 2I_yS_z + b\ I_yS_y$. The second term stays transverse on the S spin, which causes non-refocused chemical shift evolution, i.e. a cosine-modulation along ν_S between the operators $2I_yS_y$ and $2I_yS_x$. The carbon (S) evolution continues during the first half of the second refocused delay, leading to overall magnetization right before the second 180° pulse on the S spin of the form

$$a\ 2I_yS_z + b\ \cos(\nu_S\delta)\ 2I_yS_y + b\ \sin(\nu_S\delta)\ 2I_yS_x \rightarrow (a^2 - b^2\cos(\nu_S\delta))\ 2I_yS_z + ab(1+\cos(\nu_S\delta))\ 2I_yS_y + b\ \sin(\nu_S\delta)\ 2I_yS_x, \tag{S10}$$

rendering a $\cos(\nu_S\delta)$-modulation on the *intensity* of the wanted $2I_yS_z$ magnetization, where the delay δ corresponds to the sum of the second half of the first delay and the first half of the second delay. This effect directly explains the observed stripe-like behavior along the ν_S axes in Fig. S2 C,D for Φ_{PP} and $\Phi^*_{\mathrm{UR}(^1\mathrm{H})}$, as in this case only the ^{1}H subset of final operators (I_x, I_y, I_z) contributes to the fidelities, which are all modulated *in intensity* due to the imperfect inversion properties of the hard pulse applied on the S spin. This loss in intensity is also included in $\Phi^*_{\mathrm{UR}(^1\mathrm{H})}$ as a kind of point-to-point property for the inversion of S spin z-components.

Φ^*_{UR}, in contrast, describes the projection of the overall effective rotation onto the ideal desired rotation in the 16-dimensional Hilbert space of a two-spin system. This includes the rotation of ^{1}H inphase magnetization as well as rotations involving the S spin, i.e. S inphase components, but also two-spin z-order, antiphase, double-quantum, and zero-quantum operators, which all transform differently. Starting with $2I_yS_z$ antiphase, for example, will result in $-I_x$ inphase after the first half of the first refocused delay under the conditions assumed above. It will not be affected at all by the inversion properties of the hard 180° S pulse. Whenever a cosine-type modulation is seen in a quality factor with point-to-point contribution, i.e. Φ_{PP} and $\Phi^*_{\mathrm{UR}(^1\mathrm{H})}$, a purely universal rotation type quality factor will describe it as a far more homogeneous simple tilt of the rotation axis in the Hilbert space. As a result one should be aware that a reduced performance in a universal rotation quality factor usually has a much stronger effect on an element performance as an equally reduced point-to-point property. This observation is generally true, explaining why point-to-point pulses, for example, show already quite good performance with quality factors of ≈ 0.995, while for universal rotation quality factors fidelities larger than 0.9999 should be reached for comparable performance. $\Phi^*_{\mathrm{UR}(^1\mathrm{H})}$ is a special case, as it has two components comprising universal rotation as well as point-to-point properties, where the dominant effect results from the point-to-point component.

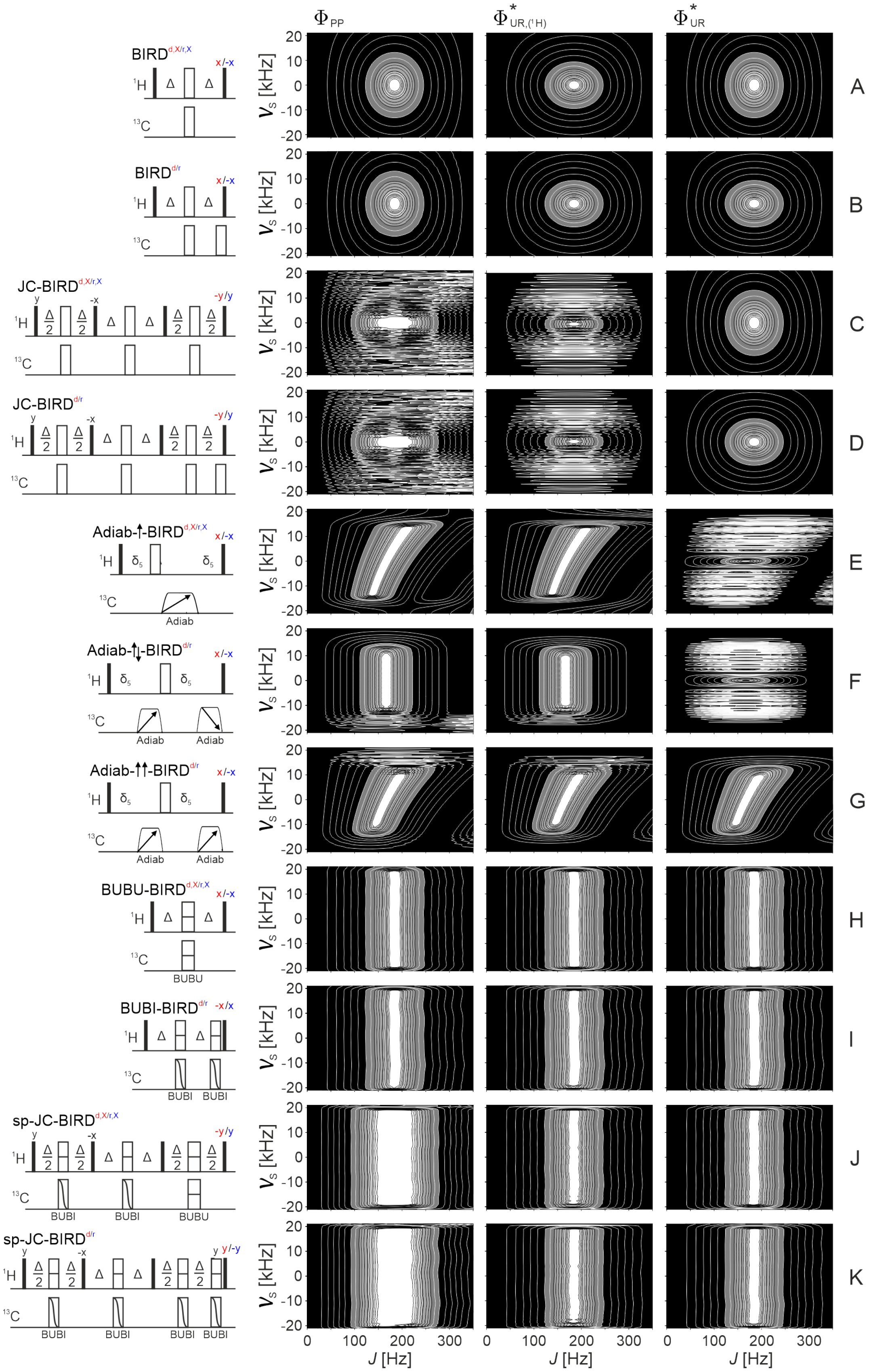

Fig. S2: Figure continues on the next page in Figure S3. See figure caption there for details.

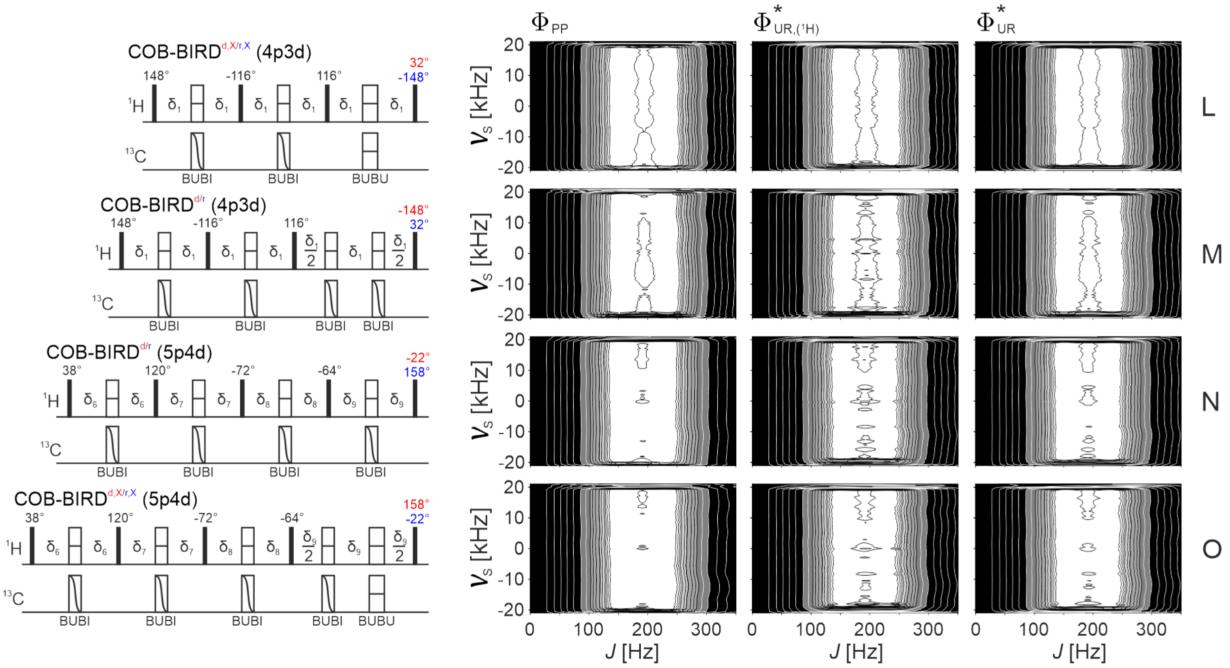

Fig. S3: **Full invetigation of all possible BIRD elements disscussed in this work on their efficacy of spin inversion, and the ^{1}H- and full universal bilinear π rotation.** Several types of BIRD elements with their BIRD^{d,X} and BIRD^{d} variants are simulated to obtain their ν_S- vs. J-dependences with respect to Φ_{PP}, $\Phi^*_{\mathrm{UR}(^1\mathrm{H})}$, and Φ^*_{UR}. In all cases, pulse sequences are given at the lefthand side, where always two different variants are indicated by the red and blue color-coding in the names and phases. Hard pulses, adiabatic pulses, inversion, and refocusing pulses are given with the same symbols as explained in the figure captions of the main article. The first four rows (A-D) represent hard pulse BIRD and JC-BIRD elements with typically low offset bandwidth in the S nucleus. Hard pulses were simulated with 25 kHz rf-amplitude. Next, three rows (E-G) with different adiabatic setups are shown, where ↑ and ↓ indicate the sweep directions of the adiabatic pulses. Φ_{PP} and $\Phi^*_{\mathrm{UR}(^1\mathrm{H})}$ exhibit very good performance. As $\Phi^*_{\mathrm{UR}(^1\mathrm{H})}$ only considers $z \to -z$ inversion properties of the heteronucleus, the performance of adiabatic pulses is essentially not compromised in applications where this is sufficient. Φ^*_{UR}, taking into account also the refocusing properties for universal bilinear rotations, is only fulfilled for the adiabatic-↑↑-BIRD sequences, where the two identical shapes compensate their complex offset-dependence. In the following two rows (H,I) offset and J-compensated BUBU and BUBI pulse sandwiches are used in the original BIRD to obtain excellent offset profiles. Same pulse shapes are then used in the next two rows (J,K) to make the JC-BIRD sequences robust against offsets. Slight changes have been applied in the BUBI-BIRD (I) and sp-JC-BIRD (K) sequences with respect to their original versions (B and D) to be able to maintain the excellent J-compensation of the BUBI pulse sandwiches. In the last four rows (L-O), $4p3d$ and $5p4d$ COB-BIRD sequences with BUBU/BUBI pulse sandwiches show essentially very high robustness against coupling, offset, and B_1 (not shown) variations in the desired ranges. Delays have been set to δ_1 = 2.583 ms, δ_6 = 2.881 ms, δ_7 = 2.036 ms, δ_8 = 0.863 ms and δ_9 = 1.969 ms, which all ensure optimal transfer for J = 120-250 Hz. The color regions indicating the fidelity ranges are chosen as black, dark grey, grey, light grey and white from -1.0 up to 0.5, 0.75, 0.9, 0.95, and 1.0, respectively and contour lines are added in white for the -0.75, -0.5, -0.25, 0.0, 0.25, 0.5, 0.65 and in black for 0.75, 0.8, 0.85, 0.9, 0.925, 0.95, 0.96, 0.97, 0.98.

Element	ρ_T	$U_T(I,J,S)$	$U_T(I,J)$
BIRD^{d,X}	$-I_z$	$\exp(-i\pi 2I_yS_y)$	$\exp(-i\pi 2I_yS_z)$
BIRD^{r,X}	$+I_z$	$\exp(-i\pi 2I_zS_y)$	$\exp(-i\pi 2I_zS_z)$
BIRD^{d}	$-I_z$	$\exp(-i\pi 2I_yS_z)$	$\exp(-i\pi 2I_yS_z)$
BIRD^{r}	$+I_z$	$\exp(-i\pi 2I_zS_z)$	$\exp(-i\pi 2I_zS_z)$
JC-BIRD^{d,X}	$-I_z$	$\exp(-i\pi 2I_xS_y)$	$\exp(-i\pi 2I_xS_z)$
JC-BIRD^{r,X}	$+I_z$	$\exp(-i\pi 2I_zS_y)$	$\exp(-i\pi 2I_zS_z)$
JC-BIRD^{d}	$-I_z$	$\exp(-i\pi 2I_xS_z)$	$\exp(-i\pi 2I_xS_z)$
JC-BIRD^{r}	$+I_z$	$\exp(-i\pi 2I_zS_z)$	$\exp(-i\pi 2I_zS_z)$
Adiab-↑-BIRD^{d,X}	$-I_z$	$\exp(-i\pi 2I_yS_y)$	$\exp(-i\pi 2I_yS_y)$
Adiab-↑-BIRD^{r,X}	$+I_z$	$\exp(-i\pi 2I_zS_x)$	$\exp(-i\pi 2I_zS_z)$
Adiab-↑↓-BIRD^{d}	$-I_z$	$\exp(-i\pi 2I_yS_z)$	$\exp(-i\pi 2I_yS_z)$
Adiab-↑↓-BIRD^{r}	$+I_z$	$\exp(-i\pi I_z)$	$\exp(-i\pi S_z)$
Adiab-↑↑-BIRD^{d}	$-I_z$	$\exp(-i\pi 2I_yS_z)$	$\exp(-i\pi 2I_yS_z)$
Adiab-↑↑-BIRD^{r}	$+I_z$	$\exp(-i\pi 2I_zS_z)$	$\exp(-i\pi 2I_zS_z)$
BUBU-BIRD^{d,X}	$-I_z$	$\exp(-i\pi 2I_yS_y)$	$\exp(-i\pi 2I_yS_z)$
BUBU-BIRD^{r,X}	$+I_z$	$\exp(-i\pi 2I_zS_y)$	$\exp(-i\pi 2I_zS_z)$
BUBI-BIRD^{d}	$-I_z$	$\exp(-i\pi 2I_yS_z)$	$\exp(-i\pi 2I_yS_z)$
BUBI-BIRD^{r}	$+I_z$	$\exp(-i\pi 2I_zS_z)$	$\exp(-i\pi 2I_zS_z)$
sp-JC-BIRD^{d,X}	$-I_z$	$\exp(-i\pi 2I_xS_y)$	$\exp(-i\pi 2I_xS_z)$
sp-JC-BIRD^{r,X}	$+I_z$	$\exp(-i\pi 2I_zS_y)$	$\exp(-i\pi 2I_zS_z)$
sp-JC-BIRD^{d}	$-I_z$	$\exp(-i\pi 2I_xS_z)$	$\exp(-i\pi 2I_xS_z)$
sp-JC-BIRD^{r}	$+I_z$	$\exp(-i\pi 2I_zS_z)$	$\exp(-i\pi 2I_zS_z)$
COB-BIRD^{d,X} (4p3d)	$-I_z$	$\exp(-i\pi 2I_yS_y)$	$\exp(-i\pi 2I_yS_z)$
COB-BIRD^{r,X} (4p3d)	$+I_z$	$\exp(-i\pi 2I_zS_y)$	$\exp(-i\pi 2I_zS_z)$
COB-BIRD^{d} (4p3d)	$-I_z$	$\exp(-i\pi 2I_yS_z)$	$\exp(-i\pi 2I_yS_z)$
COB-BIRD^{r} (4p3d)	$+I_z$	$\exp(-i\pi 2I_zS_z)$	$\exp(-i\pi 2I_zS_z)$
COB-BIRD^{d} (5p4d)	$-I_z$	$\exp(-i\pi 2I_yS_z)$	$\exp(-i\pi 2I_yS_z)$
COB-BIRD^{r} (5p4d)	$+I_z$	$\exp(-i\pi 2I_zS_z)$	$\exp(-i\pi 2I_zS_z)$
COB-BIRD^{d,X} (5p4d)	$-I_z$	$\exp(-i\pi 2I_yS_y)$	$\exp(-i\pi 2I_yS_z)$
COB-BIRD^{r,X} (5p4d)	$+I_z$	$\exp(-i\pi 2I_zS_y)$	$\exp(-i\pi 2I_zS_z)$

Table S2: **All ρ_T, $U_T(I,J,S)$, and $U_T(I,J)$ used for the calculations of Φ_{PP}, Φ^*_{UR} and $\Phi^*_{\mathrm{UR}(^1\mathrm{H})}$, respectively, presented in Figure S2 and S3**. All bilinear rotations are presented as named in Figs. S2 and 3 and their corresponding ρ_T, $U_T(I,J,S)$, and $U_T(I,J)$ used for the simulation are presented.

Type	t_p [μs]	# dig	BW [kHz]	RF [kHz]	ϑ [%]	Fidelity
$WURST_{40}$	1000	1000	20	5.6	0	0.99898
$WURST_{40}$	2000	1000	25	4.0	0	0.99920
xyBEBOP	300	300	37.5	10	5	0.99999
BEBOP(z $\rightarrow -$y)	150	150	10	20	20	0.99994
BEBOP(z $\rightarrow -$y)	700	700	37.5	10	5	0.99965
BIBOP(z $\rightarrow -$z)	200	200	10	20	20	0.99997
BURBOP 22_x	150	150	10	20	20	0.99974
BURBOP 32_x	200	200	10	20	20	0.99998
BURBOP 38_x	150	150	10	20	20	0.99977
BURBOP 64_x	150	150	10	20	20	0.99978
BURBOP 72_x	150	150	10	20	20	0.99979
BURBOP 90_x	200	200	10	20	20	0.99997
BURBOP 90_x	700	700	37.5	10	5	0.99946
BURBOP 116_x	200	200	10	20	20	0.99997
BURBOP 120_x	200	200	10	20	20	0.99996
BURBOP 136_x	800	800	37.5	10	5	0.99970
BURBOP 148_x	200	200	10	20	20	0.99995
BURBOP 158_x	200	200	10	20	20	0.99993
BURBOP 180_x	1000	1000	37.5	10	5	0.99990
BUBI(^{1}H)	600	1200	10	20	20	0.99998
BUBI(^{13}C)	600	1200	37.5	10	5	0.99914
BUBU(^{1}H)	1000	2000	10	18.5	20	0.99998
BUBU(^{13}C)	1000	2000	37.5	20	5	0.99991

Table S3: **All parameters related to each shaped pulse used in the different BIRD, Adiabatic-BIRD and COB-BIRD sequences and the COB3-INEPT implemented in the refocussed INEPT-type experiments.** For each shape, in order, the pulse time (t_p), total number of digits (# dig) – i.e. the number of individual elements with constant phase and amplitude of the digitized shaped pulse –, the controlled bandwidth (BW), maximum amplitude (RF), B_1-compensation (ϑ) and the corresponding fidelity are presented. For the different shaped pulse nomenclatures see Table 2 in the main text.

4 Optimization of TOP-curves for different BIRD variants

To obtain a time optimal (TOP)-curve characterizing the physical limits of BIRD elements, Φ_{UR} was implemented for a heteronuclear two-spin system using the four different pairs of target propagators given in Table 1 in the main article.

First, shaped pulses were optimized with a rather coarse digitization of 100 μs to define an upper threshold for Φ_{UR} for a hard pulse-delay approximation considering only the desired J-dependence of the filter element (38, 39). Ten optimizations with different starting pulses are used to find the optimal sequence for any given overall pulse length between 0.5 and 25 ms incremented in steps of 500 μs. The best sequence of each set then contributes to the TOP-curve. However, even if protons are assumed to be on-resonance, the optimization of BIRD elements is not as straightforward as with other reported transfer elements (38, 39). Given that BIRD^{d,X} and BIRD^{r,X} filters need to manipulate the S spin, the corresponding propagators cannot be created without concurrent RF irradiation on both spins. Even BIRD^d and BIRD^r filters require pulses on spin S in cases where ϕ_2 differs from ϕ_4 since this entails occasional composite rotations with a monolinear spin S contribution that cannot be created without RF pulses on the S spin. Bearing this in mind, pulse sequences that are tolerant to a typical range of $^1J_{CH}$ couplings of 120-250 Hz for hybridizations could be obtained. The resulting TOP curves for all BIRD variants of Table 1 are overlaid in Figure S4. The TOP-curve derived for BIRD^d overall shows the best quality factors and is used as the "Shape" curve in Figure 3 in the main article.

Since all basic BIRD sequences have the same structure, it can be assumed that the underlying mode of action is the same for all BIRD variants and thus the GRAPE algorithm can find similarly optimal solutions in each case. Therefore all TOP curves should lay on top of each other if good convergence is assumed. They more or less do with a single exception of the BIRD^r element where ϕ_2 differs from ϕ_4 (RF controls on spin S needed) and an effective propagator corresponding to unity with an imaginary phase factor has to be created for the directly-bound protons. This particular TOP curve only follows the others up to a first substantial dip around 5 ms and then hardly ever reaches the level where all other curves are clustered again. This notable exception may not be attributed to convergence issues alone but might be due to the fact that spin systems with a range of J-couplings cannot be left unstirred to the same extent as demanded by this particular set of target propagators.

The three distinct dips in the majority of the TOP curves in the logarithmic representation are a familiar phenomenon in exploring the limits-type optimization studies, where beyond a certain threshold in pulse length a new family of pulse shapes is made available (40, 47). Just as phase modulation can create the effect of a second irradiation frequency, BIRD sequences of a certain length can behave as if they match two or more distinct J-couplings.

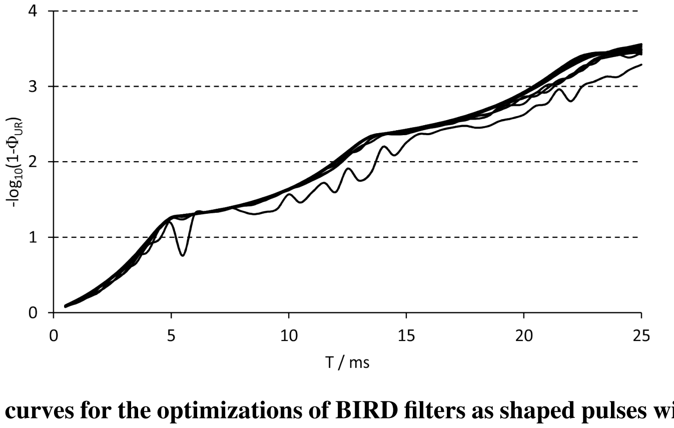

Fig. S4: **Interpolated TOP curves for the optimizations of BIRD filters as shaped pulses with effective propagators as given in Table 1 in the main article.** Fidelities are given on a negative logarithmic scale of the error functional $1-\Phi_{UR}$ to show a rather monotonous increase.

5 Hard Pulse Delay TOP-curve optimizations and experimental verification

The continuous shapes obtained so far are exclusively compensated against a variation in heteronuclear J-couplings. The next step towards sequences which are robust against J-couplings, offsets and B_1-inhomogeneities is the hard pulse-delay approximation (38, 39). Continuous pulse shapes are approximated by a set of hard pulses and evolution delays with varying degrees of complexity. The specific pulse sequence used for optimizations in the following is a BIRD^d element from Table 1, which uses phases ϕ_1 to $\phi_4 = x$ and facilitates a π rotation around the $2I_y^d S_z$ axis, and the unity operation on remotely-bound protons. Given that the net rotation on ^{13}C amounts to 360°, the pulse sequences can be optimized without RF pulses on ^{13}C. For the optimization of hard pulse-delay sequences it is necessary to obtain gradients of propagators with respect to time since a set of optimal delays is required in addition to optimal flip angles. These are easily derived to

$$\frac{\partial U_j}{\partial \Delta t_j} = -i\mathscr{H}_j U_j \tag{S11}$$

and gradients of Φ_{UR} can be obtained via

$$\frac{\partial \Phi_{\mathrm{UR}}}{\partial \Delta t_j} = -\Re\langle U_{j+1}^{\dagger} \cdots U_N^{\dagger} U_T | i\mathscr{H}_j U_j \cdots U_1 \rangle. \tag{S12}$$

Again, ten optimizations with different starting pulses are used to find the optimal sequence for any given overall pulse length between 0.5 and 25 ms incremented in steps of 500 μs. The best sequence of each set will then contribute to the TOP curve. Given that the complexity of pulse sequences with a few pulses and delays is very reduced, the hypersurface of Φ_{UR} is heavily jointed so that optimizations using conjugate gradients can end up stuck in local extrema very quickly. This is why steepest ascent with constant values for ε were performed using $\varepsilon_{\Delta t} = 10^{-7}$ and $\varepsilon_u = 10^{10}$. These values were found semiempirically given that the length of the hard pulses is set to Δt = 0.5 μs and arbitrary RF amplitude is allowed to facilitate any flip angle while delays are in the range of a few ms. Both values for the two variables were picked to achieve a substantial change in the corresponding controls in each iteration so that optimizations can also bypass local extrema (41). Optimizations were aborted when the change in Φ_{UR} between two iterations was less than 10^{-10}. TOP curves for pulse sequences which facilitate BIRD^d rotations are given in Figure S5, which is identical to Figure 3 in the main article. The continuous pulse shape obtained for the shaped BIRD^d element defines the upper threshold for the achievable fidelities. To provide optimal transfer for J-couplings in the range of 120-250 Hz, conventional BIRD elements need to have delays calibrated to match 185 Hz. If the sequences depicted in Figure S1 are set up accordingly, the standard BIRD sequence (τ = 5.4 ms) and the JC-BIRD sequence with improved inversion properties (τ = 10.8 ms) both yield fidelities of approximately 0.945 and are included in Figure S5. Hard pulse-delay sequences with $(n+1)$ pulses and n delays are labeled as $(n+1)pnd$. It should be noted that optimizations are obtained on-resonant only, making the application of refocussing pulses during delays obsolete.

The resulting TOP curves can be interpreted rather easily. Up to the length of a conventional BIRD sequence, which corresponds to a *2p1d* sequence, all TOP curves overlap. This threshold marks the first dip in the curve of the shaped pulses which has to correspond to a simple sequence roughly calibrated to match J = 185 Hz. Also *3p2d* sequences can not exceed the fidelity of such a simple sequence. The second bulge in the TOP curve of shaped pulses marks the point where the sequences are effectively matched to two J-couplings at the same time which lie favorably within the desired range of 120-250 Hz. *4p3d* sequences have the same complexity as the JC-BIRD sequence, but can approach the threshold of roughly 0.996 fidelity at overall pulse lengths of about 15 ms. This fidelity is desirable since it also yields Φ_{PP} fidelities of about 0.99. Starting with *5p4d* sequences, the second threshold can be exceeded but at higher pulse lengths the TOP curves starts to scatter and interpolation is no longer meaningful, which may be attributed to convergence issues.

For practical applications, sequences with as little complexity as possible are desirable. Thus, only *4p3d* and *5p4d* sequences are discussed in the following, which both approach the fidelity of continuous shapes the closest at T = 15.5 ms. The best candidates for both implementations are given in Figure S6.

The analysis of the chosen pulse sequences hold certain subtleties. It has to be noted that phases are uniformly x which will be discussed further below. To construct the COB-BIRD^d, COB-BIRD^r, COB-BIRD^{d,X}, and COB-BIRD^{r,X} elements, an explanation is given in the caption of Figure S6. The sequences can be understood on a first glance from the perspective of the ^{12}C-bound protons (^{1}H^{r}), since the sum of all flip angles is either 0° or an integer multiple of 180°, and from the perspective of the inversion properties of the ^{13}C, as in the main text it is shown that this correlates nicely with the UR bilinear rotation properties of the sequence. It can easily be proven that pulses with flip angle α >180° can be replaced by pulses with a flip angle $\alpha - 360°$ to reduce the overall flip angle which will provide an inherent robustness against resonance offsets and B_1-inhomogeneities. In order to compensate transfer elements against resonance offsets, pairs of 180° pulses are inserted in the middle of magnetization transfer periods. However, these additional spin flips

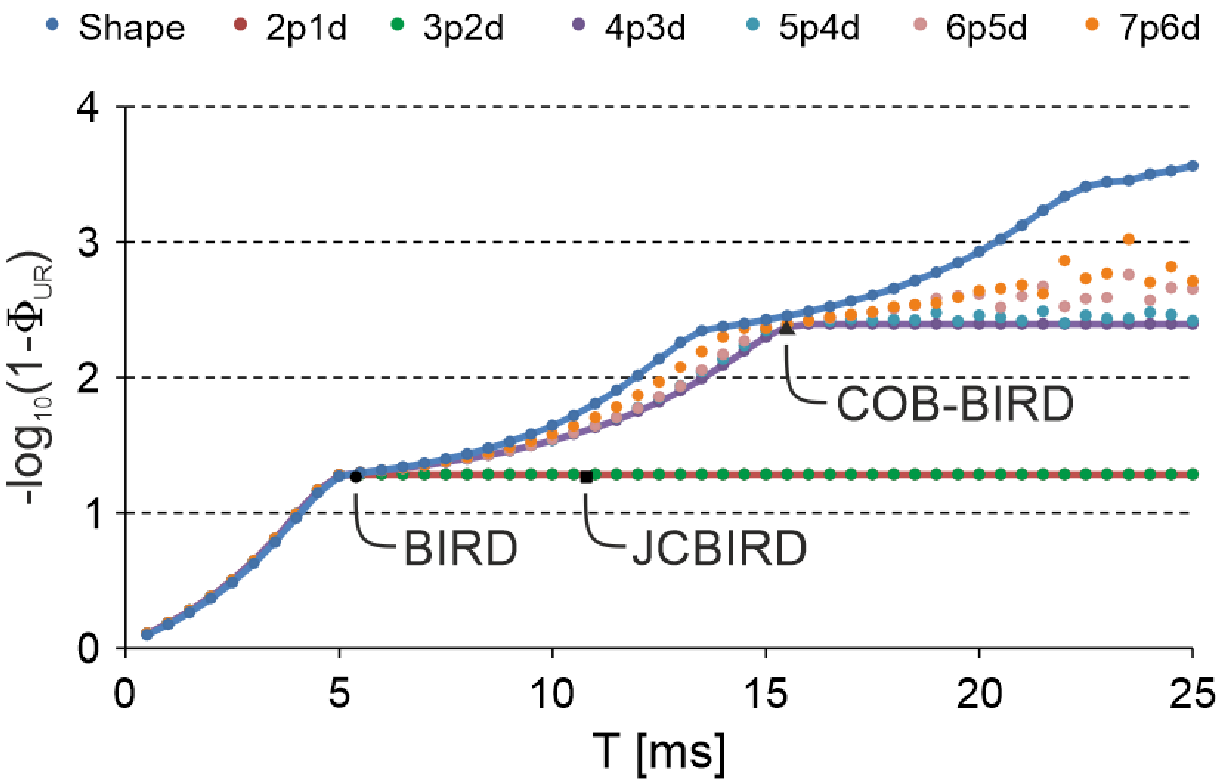

Fig. S5: **TOP curves for the optimizations of BIRD^d filters as shaped pulses and hard pulse-delay sequences.** The figure is identical to Figure 3 of the main article, but is enlarged. Fidelities are given on a negative logarithmic scale of the error functional $1-\Phi_{UR}$ to show a rather monotonous increase. The legend describes the various sequence types consisting of $(n+1)$ pulses separated by n delays. The performance of the original BIRD as well as the JC-BIRD with delays calibrated to match 185 Hz are also depicted. Fidelities are given on a negative logarithmic scale of the error functional $1-\Phi_{UR}$.

count towards the overall rotation. In the case of the COB-BIRD^{r,X} (4p3d) sequence a total 540° rotation is introduced on both ^{13}C and ^{1}H^{r} corresponding to an effective 180° rotation with respect to their initial state. Adjusting the θ_1 by +180° will therefore return the ^{1}H^{r} to its initial state and therefore the COB-BIRD^{d,X} is designed with a 540° and 360° rotation on ^{13}C and ^{1}H^{r} respectively. Introducing the additional refocussing pulse into the last transfer delay as presented in Figure S6 B, will, with respect to the COB-BIRD^{r,X} (4p3d), transform the COB-BIRD^{r,X} into its COB-BIRD^{d} counterpart, as the overall rotation for the ^{13}C and ^{1}H^{r} is increased to 720° for both, where the θ_1 can again be adjusted by +180° to achieve a 540° rotation on ^{1}H^{r} and thus designing the COB-BIRD^{r}. A similar scheme can be applied to the *5p4d* sequence to gain all the possible bilinear rotation elements. The ^{1}H^{r} in the COB-BIRD^{d}, COB-BIRD^{r}, COB-BIRD^{r,X}, and COB-BIRD^{d,X} have an overal rotation of 720°, 900°, 1080° and 900°, respectively, whereas the ^{13}C has a 720° and a 900° rotation for the COB-BIRD^{d} and COB-BIRD^{r} and the COB-BIRD^{d,X} and COB-BIRD^{r,X}, respectively. This maintenance of the rotation properties is also the reason why all phases have to be purely x (or y for that matter) because the heteronuclear transfer is facilitated by pulses with a mixed phase will be fundamentally impaired as soon as 180° pulses with pure phase are inserted.

Fidelities according to Φ_{PP} and Φ_{UR} of the proposed COB-BIRD elements were evaluated by simulations given in Figure S7. It becomes clear that not only inversion properties are improved compared to the sequence proposed in (4), but also the overall rotation can be made robust against a variation in heteronuclear J-couplings by both sequences. Within the desired range of J-couplings between 120-275 Hz the COB-BIRD (4p3d) sequence provides excellent transfer efficiency. The sequence was tested in a simple proxy setup for CLIP/CLAP-RESETHSQC experiments (21) on a sample of 140 mM sodium acetate-2-^{13}C ($^{1}J_{CH}$ = 125.3 Hz) dissolved in a 1:5 (v/v) mixture of D_2O/DMSO-d_6. Given that a ^{13}C-enriched material is used, the HSQC transfer could be replaced by a simple 90° excitation pulse as presented in Figure S8 A. Further, no homonuclear couplings are active in sodium acetate so that intensities of simple spin echoes can be evaluated after the sign of transversal magnetization was inverted by the BIRD elements. Signals of residual ^{12}C-containing material could have been cycled out using difference spectroscopy(4, 6), but the intensity profiles would

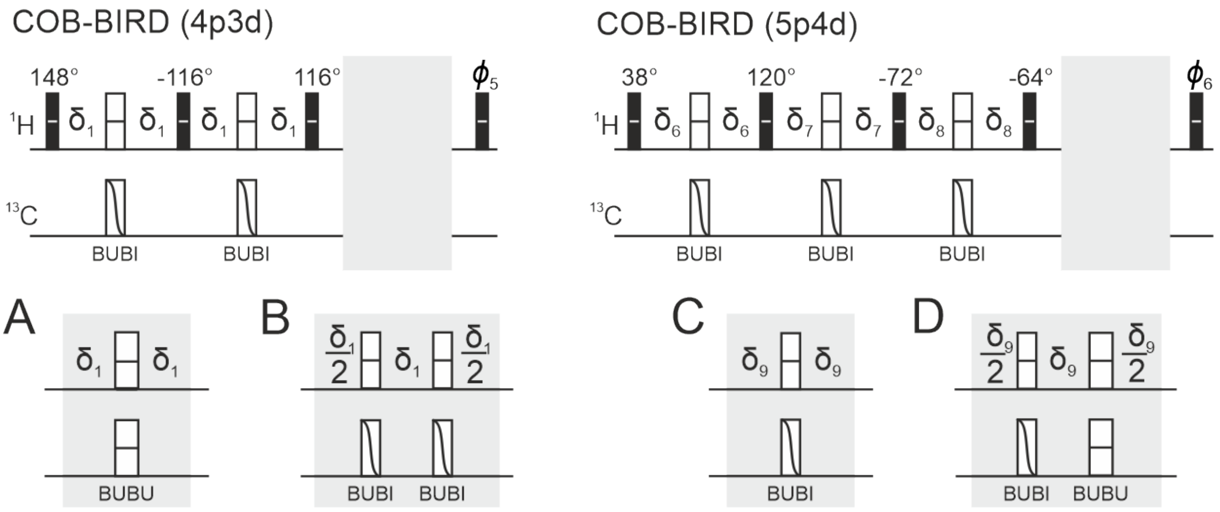

Fig. S6: **Toolkit for the construction of all four possible COB-BIRD bilinear rotation elements as the *4p3d* or the *5p4d* variant.** Combining the *4p3d* sequence with A provides hetronuclear inversion and adjusting ϕ_5 to either -148° or 32° constructs the COB-BIRD^{d,X} or COB-BIRD^{r,X}, respectively. Combining the *4p3d* sequence with B returns the heteronucleus to its initial position and adjusting ϕ_5 to 32° or -148° constructs the COB-BIRD^{d} or COB-BIRD^{r}, respectively. For the *5p4d* sequence, replacing the gray box with C and adjusting ϕ_6 to -22° or 158° constructs the COB-BIRD^{d} and COB-BIRD^{r}, respectively. Replacing the gray box with D and adjusting ϕ_6 to 158° or -22° will construct the COB-BIRD^{r,X} and COB-BIRD^{d,X}, respectively. Shaped pulses are presented as shown in Figure 2 of the main text with BUBI and BUBU pulse sandwiches as noted below the pulse shapes. All shaped pulses are used as presented in Table S3. Delays are set to δ_1 = 2.583 ms, δ_6 = 2.881 ms, δ_7 = 2.036 ms, δ_8 = 0.863 ms and δ_9 = 1.969 ms, which all ensure optimal transfer for J = 120-250 Hz.

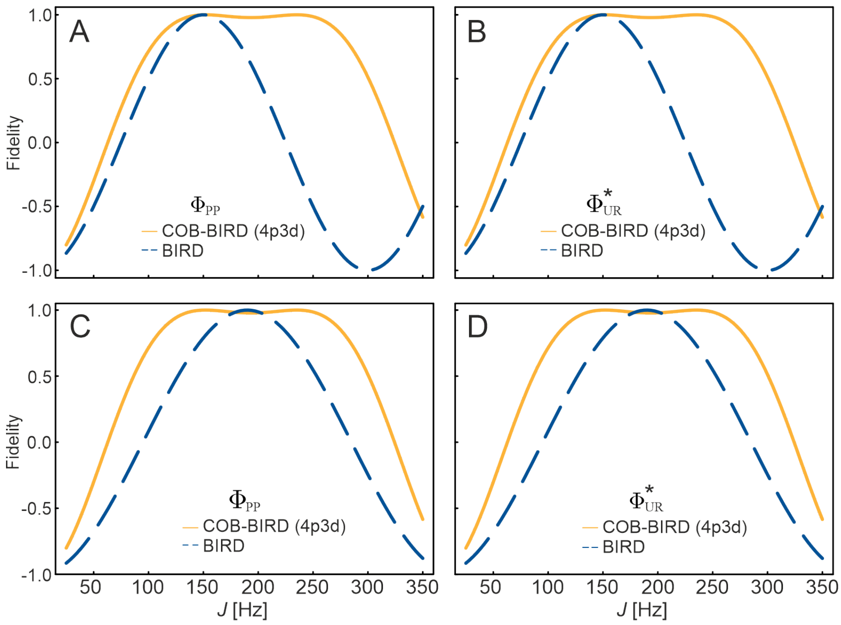

Fig. S7: **Fidelities of the BIRD (dashed blue) and COB-BIRD (solid orange) elements as a function of *J*-coupling related to their spin inversion (Φ_{PP}, A, C) efficacy and the synthesis of the desired propagator (Φ^*_{UR}, B, D).** Simulations were performed with the BIRD^{d,X} and the COB-BIRD^{r,X} as representative examples (as depicted in Figure S1 and S6) using perfect on-resonant pulses. The BIRD delays were matched to a *J*-coupling of 150 Hz or 190 Hz for A and C respectively.

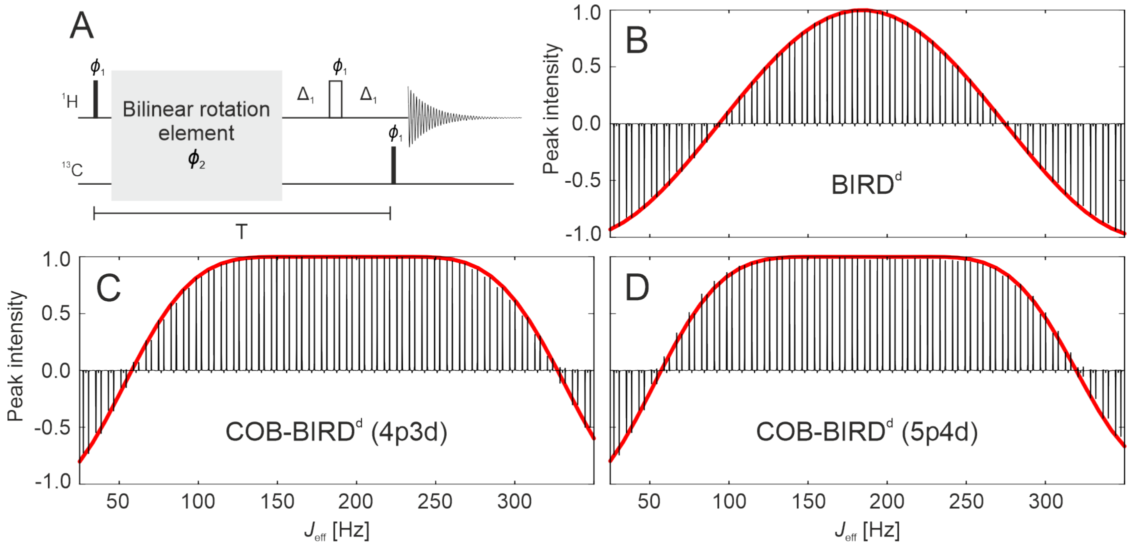

Fig. S8: **Experimental and theoretical comparison of the bilinear universal π-rotation of the BIRD and COB-BIRD elements.** Using the pulse sequence as presented in A, the BIRD^d and the 4p3d and 5p4d variants of the COB-BIRD^d fidelities are demonstrated and compared to simulations. For the bilinear rotation elements tested, the delays within the sequences are scaled as explained in the text. The bilinear rotation is followed by a variable spin echo to keep the full sequence at a constant time T. Δ_1 is set as $T/2 - \Delta_{BIRD}$, where Δ_{BIRD} is the total delay time of the scaled bilinear rotation delay. For the BIRD^d, the COB-BIRD^d (4p3d) and the COB-BIRD^d (5p4d) sequences T has been set to 15.1 ms, 44.0 ms, and 44.0 ms, respectively. The sequence was applied with the following phase cycling: ϕ_1 = 0 2 2 0 1 3 3 1. ϕ_2 = 1 3 3 1 2 0 0 2. ϕ_{rec} = 0 0 2 2 1 1 3 3, where ϕ_2 is applied for all pulses in the bilinear rotation element equally and ϕ_{rec} is the aqcuisition phase.

be distorted compared to the simulations because the second scan would have full intensity in each case due to the refocusing of heteronuclear couplings by the 180° ^{1}H pulses within the BIRD elements. A comparison between simulated and experimental BIRD^d spin echo intensity profiles is given in Figure S8 B, for which effective J-couplings were used in the experiment as explained in the following.

Applying the procedure described in (38), the value for J_{eff} has been varied in the experiments by applying scaling factors to delays calibrated to match 185 Hz in the case of conventional BIRD according to

$$\Delta_{eff} = \frac{J_{eff}}{2J_{del} \cdot J_{exp}} \tag{S13}$$

with J_{del} = 185 Hz and J_{exp} = 125.3 Hz, where J_{exp} is the actual coupling constant of the compound used. Delays for the COB-BIRD sequences given in Figure S6 were scaled accordingly by J_{eff}/J_{exp}. For the simulations and experiments 41 values for J_{eff} were sampled between 25 and 350 Hz. Residual antiphase contributions to the observed signal were removed by a hard 90° pulse on ^{13}C prior to acquisition as shown in Figure S8 A. Since the length of the sequences varies substantial between the extreme values of J_{eff}, relaxation losses could be observed during preliminary experiments on a sample with a reduced T_2 time due to doping with a paramagnetic relaxation agent. Thus, a variable echo period was appended to the BIRD elements so that the overall relaxation period was kept constant at T = 15.1 ms, 44.0 ms and 44.0 ms for the BIRD^d, and the *4p3d* and *5p4d* COB-BIRD^d sequences respectively. Signal intensities were normalized to the maximum peak intensity which was achieved across the given range of J-couplings.

All experimental profiles show excellent agreement with the simulations. Small negative peaks can be observed at the midpoints of each heteronuclear doublet which correspond to signal of the residual ^{12}C-containing acetate. Since the signal is phased to show the inverted signal of ^{13}C-bound protons with positive intensity and the ^{12}C-bound protons were left untouched by the BIRD^d rotation, the latter appear negative. All intensity profiles are meaningful in the sense of showing maximum intensity either at $J_{eff} = J_{del}$ for conventional BIRD or within the optimized range of 120-250 Hz for the coupling compensated BIRD versions while the maximum negative intensity is approached but not reached for the minimum value of J_{eff} = 25 Hz. Further, the profile of the *4p3d* is slightly more homogeneous than for the *5p4d* sequence. This can be attributed in parts to the fact that the latter is using more ^{1}H pulses and is thus more susceptible to B_1-inhomogeneities and miscalibration given that hard pulses were used. Moreover, the inherent structure of the *4p3d*

is more advantageous than its *5p4d* counterpart. It has the beneficial symmetry properties described in (49) given that all transfer delays have equal duration and the second half of the sequence is the time and phase-reversed version of the first half. UR pulses with inherent symmetries with respect to the control-amplitudes have also been found to be advantageous in (40). For the simulation, ideal on-resonant hard pulses were used together with the quality factor Φ_{PP} ($-I_x \rightarrow I^-$) (See Eq. 2 of the main text).

6 Refocused 2D-COB3-INEPT with ω_1-BIRD^r decoupling

Similar to the *J*-resolved type refocused 2D-COB3-INEPT experiments comparing the efficacy of the BIRD^{d,X}, JC-BIRD^{d,X} and the COB-BIRD^{d,X} regarding their refocussing properties, an experiment was conducted to compare the BIRD^r and the COB-BIRD^r experiments where both chemical shift and $^1J_{CH}$ are retained while decoupling homonuclear long range couplings in a ω_1-COB3-INEPT experiment as presented in Figure S9. The experiment is excecuted as presented in the main text, where only the gradient G_4 is inverted as the measured spin is left untouched in the BIRD^r and COB-BIRD^r sequences. As the reference (Figure S9 A), a free evolution period is maintained in the t_1 delay. The BIRD^r delays were again matched to 125 Hz. Comparing the reference sequence to the BIRD^r decoupled sequence it can be seen that, similar to the *J*-resolved case, the **6** and **4** signals are decoupled and show a S/N increase of 3.4x and 1.5x respectively. The total couplings $^1T_{\mathbf{2}}$ and $^1T_{\mathbf{5}}$ of 253 Hz and 243 Hz, respectively, are clearly outside the range of the BIRD^r elements to effectively retain $\Phi^*_{UR(^1H)}$, which resulted in uninterpretable patterns with artifacts and low S/N. In the case of the COB-BIRD^r the experiment shows excellent S/N for all $^1T_{CH}$ values in the partially aligned (-)-nicotine sample with a S/N increase of 4.7, 3.8, 1.3, and 6.2 times with respect to the reference experiment for the $^1T_{\mathbf{2}}$, $^1T_{\mathbf{6}}$, $^1T_{\mathbf{4}}$, and $^1T_{\mathbf{5}}$ couplings, respectively, where the $^1T_{CH}$ values are found at 252.5, 116.7, 71.5, and 242.9 Hz, respectively.

The COB-BIRD^r as a homonuclear selective refocussing element with respect to free evolution has been tested on the full spectral width of the partially aligned (-)-nicotine sample as presented in Figure S10. For this experiment the sequence as presented in the main text has been adapted to use all shaped pulses which are shown in Table S3. The shaped pulse adapted COB-BIRD^r element used in the experiment is presented in Figure S10 B. cross-sections in the F1 dimension for each signal in (-)-nicotine are presented with their respective S/N in Figure S11. It can be seen that all CH signals (**2**, **6**, **4**, **5**, and **8**) show excellent homonuclear decoupling with substantial S/N increase for both the high $^1T_{CH}$ of **2** and **5** at 253 and 243 Hz respectively and for the low $^1T_{CH}$ of **4** at 71 Hz. The main challenge of the BIRD and their variants lay in the fact that for CH_2 and CH_3 groups spin systems with two or three directly bound protons are being manipulated equally, which essentially means no effective $^2J_{HH}$ decoupling should occur. For the CH_3 group in (-)-nicotine (**12**) it can easily be seen that the $^2D_{HH}$ coupling leading to the triplet remains, but the long range homonuclear decoupling leads to substantial S/N increase as the linewidth has decreased. For the CH_2 groups in **11**, **9**, and **10**, S/N increase is not so substantial. Suprisingly, the **11** CH_2 protons show good homonuclear decoupling with a S/N increase of 1.2x and 1.5x for the signals with $^1T_{CH}$ of 97 Hz and 170 Hz, respectively. The **9** and **10** CH_2 groups show substantial strong coupling artifacts within the group as well as with the neighboring CH_2 groups, leaving the distorted signals very difficult to interpret. For the **10** proton multiplets similar $^1T_{CH}$ values can still be found of 152 and 134 Hz, whereas the slice of **9** has become uninterpretable.

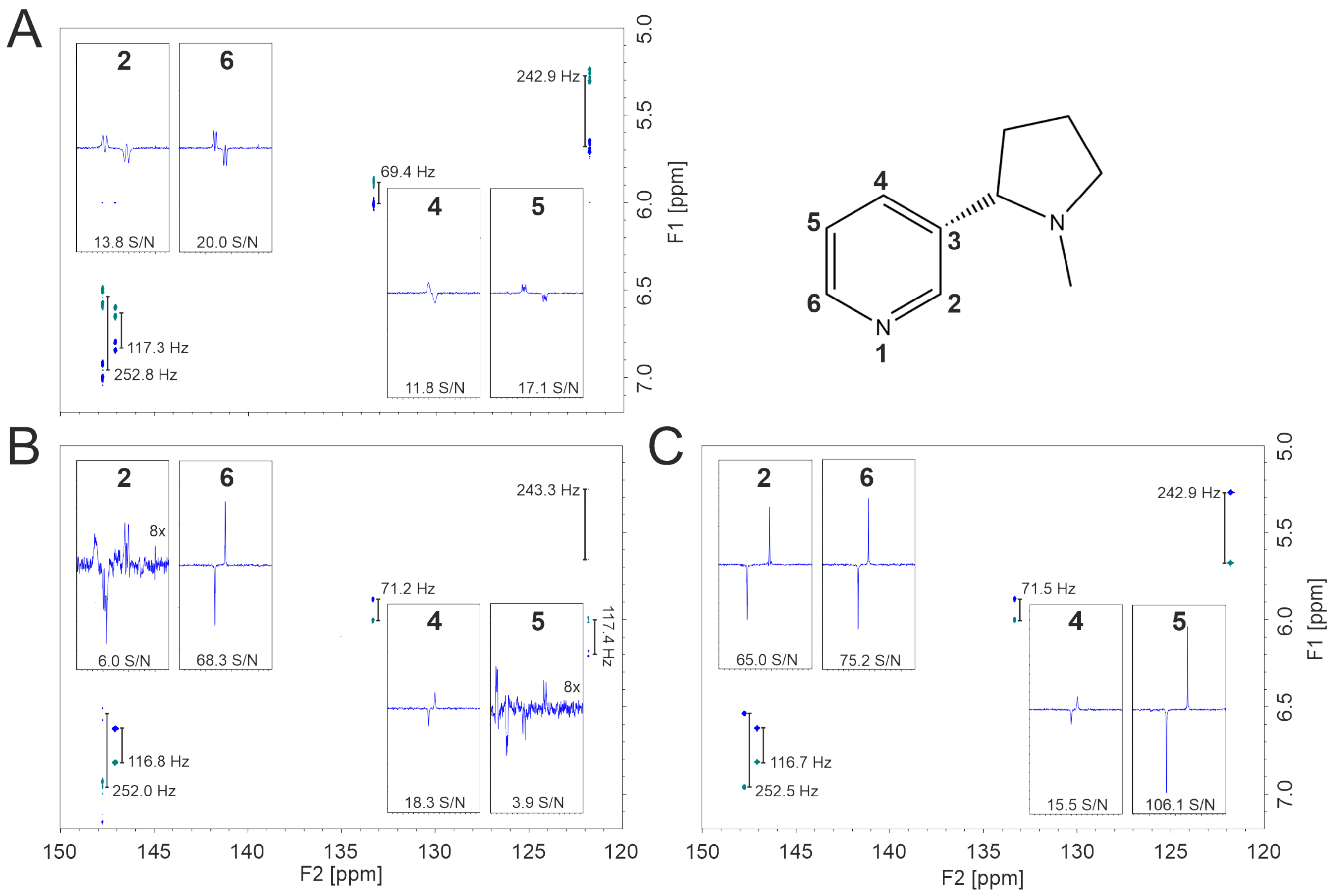

Fig. S9: **Experimental comparison of various refocussing elements applied in a ^{13}C-detected, ^{1}H-decoupled, ω_1-INEPT experiment.** The sequence was used as presented in Figure 6 of the main text, where only G_4 is sign inverted as, in contrast to the BIRD^{d,X} variants, the BIRD^{r} does not refocus the directly bound protons. The aromatic numbering of (-)-nicotine is presented at the top right of the figure. For the reference (A) the incrementation delay is left as a free evolution. For each experiment, slices of the aromatic signals are extracted along the F1 dimension and shown with the corresponding measured T-coupling and S/N. The transfer delays in the BIRD^{d,X} and JC-BIRD^{d,X} are set to 125 Hz and the COB-BIRD^{d,X} is applied as presented in Figure S6.

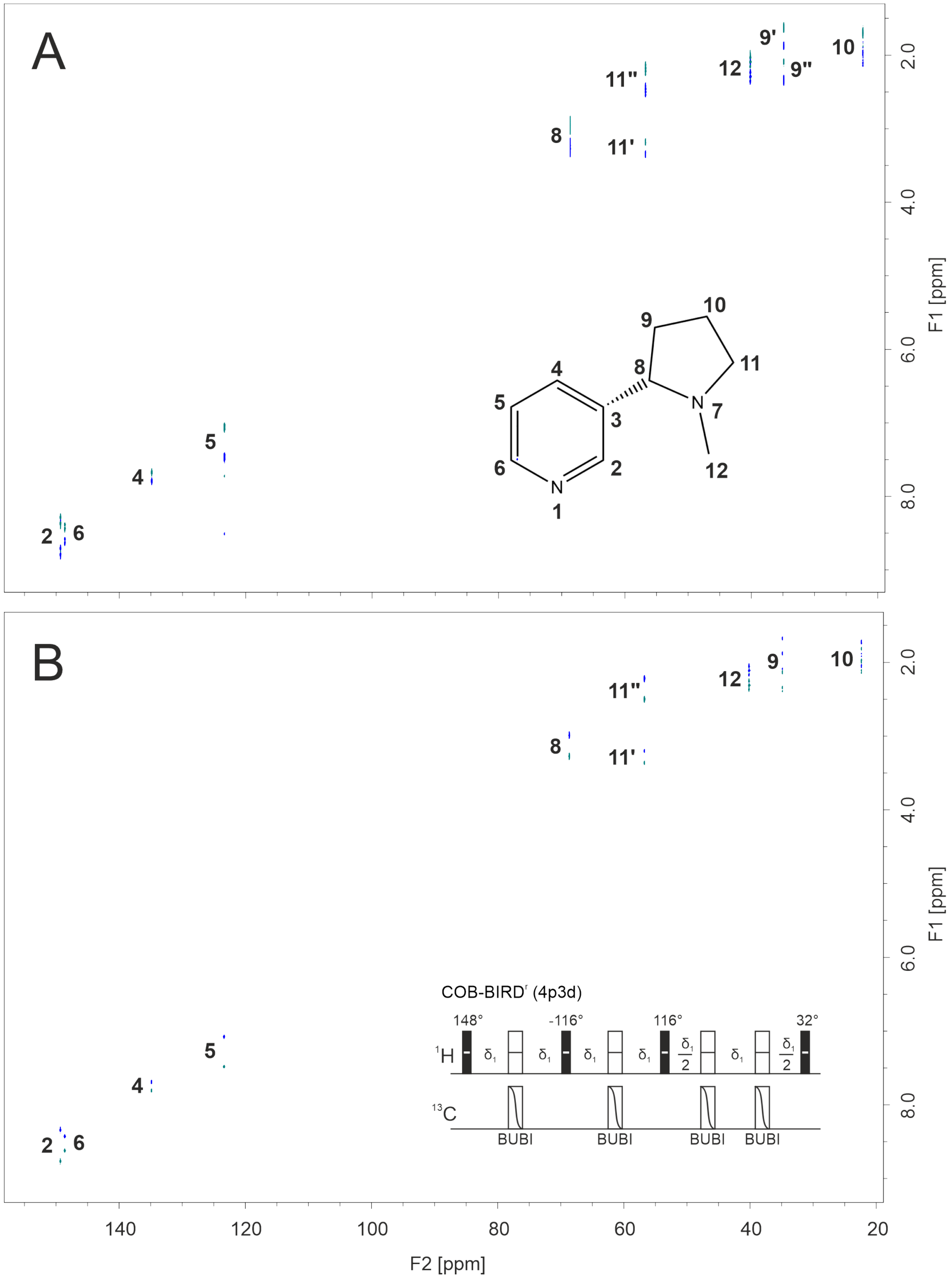

Fig. S10: **The ω_1-COB3-INEPT experiment using a BIRD and COB-BIRD selective refocusing element is applied to the full spectral width of partially aligned (-)-nicotine.** The full pulse sequence was adapted for shaped pulses as presented in Tab. S3 and the COB-BIRD^r sequence used is presented in B. The full assignment used for (-)-nicotine is presented in A and the signals are assigned accordingly. The F1 slices of each signal are presented in Fig. S11. The delay δ_1 = 2.583 ms and the black rectangles with a white dash indicate universal rotation pulses optimized for the specified flip angles (see Tab. S3)

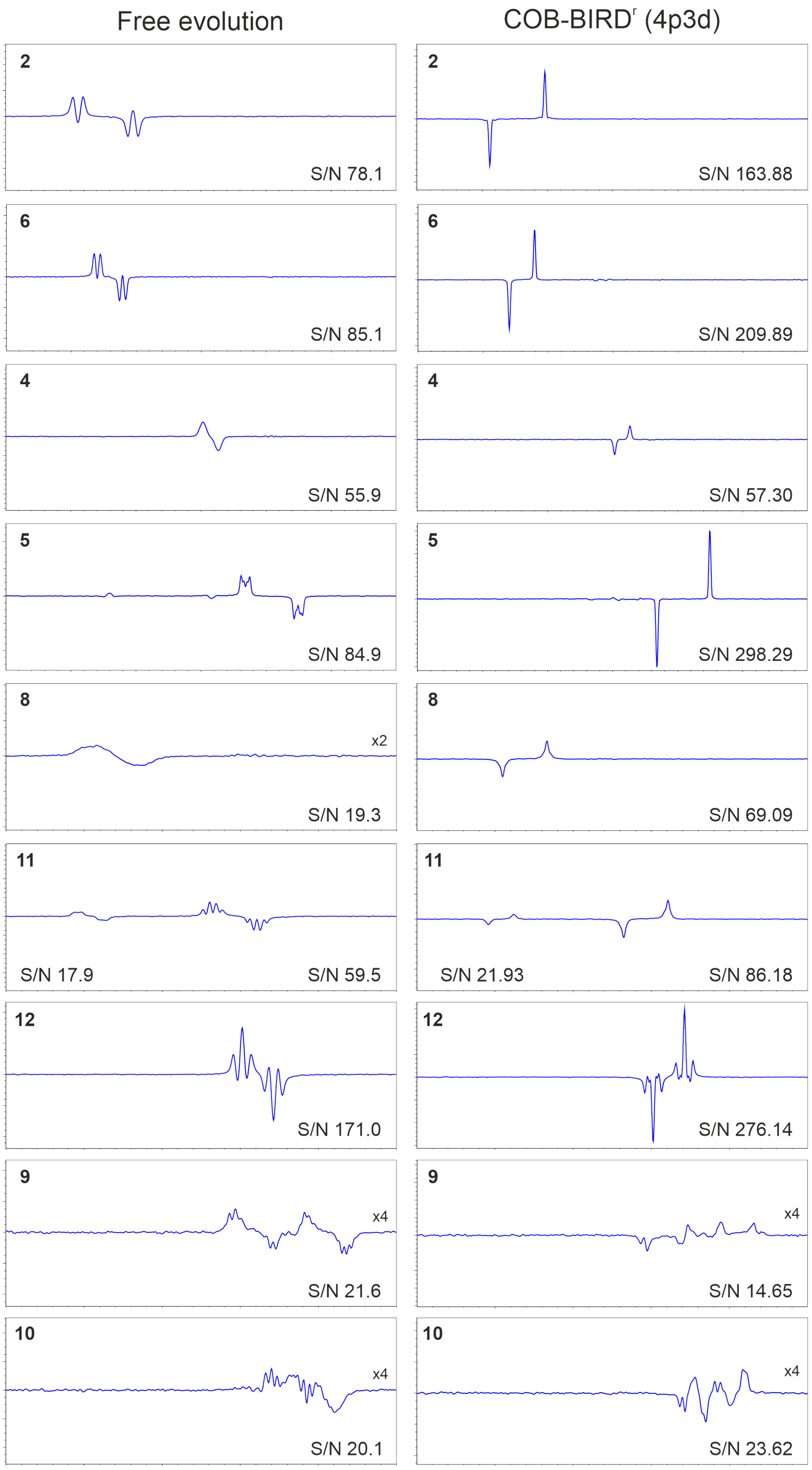

Fig. S11: **F1 slices of the ω_1-COB3-INEPT experiment as presented in Fig. S10.** Each F1 slice has been assigned on the top left according to the assignment presented in the 2D spectrum. The measured S/N has been shown on the bottom of the slice.

7 Details on the COB-BIRD^{d,X} experiment and its application to a 11-mer peptide

For a more detailed look on the homonuclear decoupling of the COB-BIRD^{d,X} element presented in Fig. 6 of the main text, the spin echo and the COB-BIRD^{d,X} variants are directly compared in Fig. S12. The comparison shows the simplification gained from implementing the COB-BIRD^{d,X} as the complex multiplicity structures are reduced to a single doublet - corresponding to the heteronuclear $^{1}T_{CH}$. Besides the increase in S/N as is presented in the main text, the decoupling also severely enhances the reliability of extracting single bond heteronuclear couplings. In order to give a better understanding of the possibilities of the COB-BIRD^{d,X} filtered J-INEPT sequence, it has been applied to a 11-mer peptide (Fig. S13). The pulse sequence has been applied to the $^{13}C_{\alpha}$ CH-group with both the spin echo refocussing (blue-green contours) and COB-BIRD^{d,X} selective refocussing (purple-red contours). The results of this experiment show excellent decoupling of the homonuclear J-coupling. In Tab. S4 and S5 the corresponding relaxation times are shown for both the aligned (-)-nicotine sample as well as the peptide, respectively. In Fig S14 the full pulse sequences for the application of the refocussed COB-BIRD experiments are presented. The COB-BIRD^{d,X} (A) filter and COB-BIRD^{r} (B) are implemented as applied in Figs. 6 and S9, respectively, and use pulsed field gradients for coherence selection. Alternatively, the sequence can be adapted to employ a phase program for coherence selection (C). In this case, only a single purge gradient is applied as a z-filter for spectral cleanup.

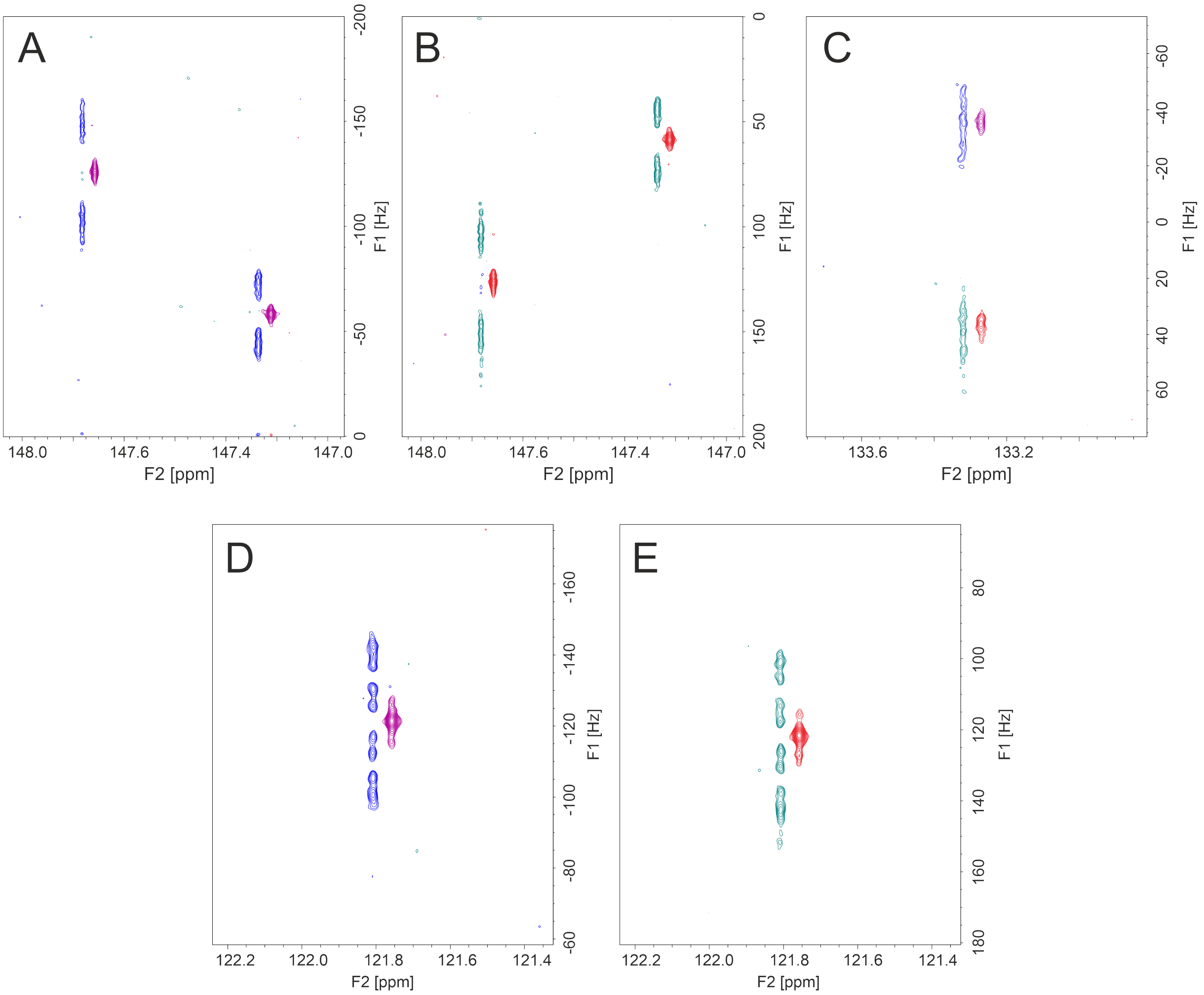

Fig. S12: **Direct comparison of the 2D line contours with an expansion of the Figs. 6 B and 6 E as the conventional spin echo and the COB-BIRD^{d,X} selective refocussing, respectively.** The blue and green contour lines show the spin echo version and the purple and red show the COB-BIRD^{d,X} version. Following the assignment as presented in Fig. 6, A and B show **2** and **6**, C shows **4** and D and E show cross peaks for **5**. The COB-BIRD^{d,X} cross peaks have been shifted for visibility.

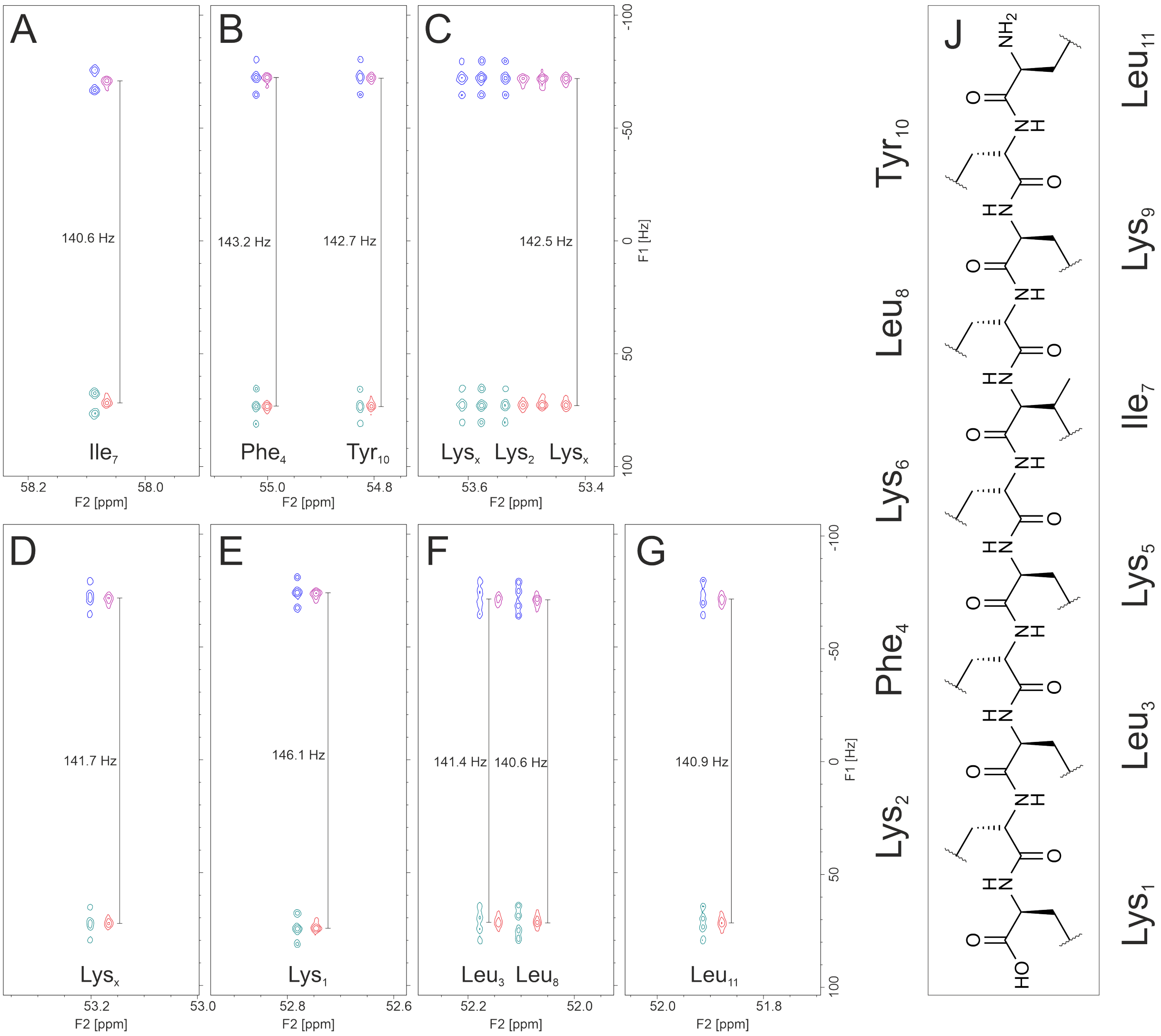

Fig. S13: **Experimental validation of the COB-BIRD selective refocusing for indirect decoupling on an 11-mer peptide.** Experimental comparison of the ^{13}C-detected refocussed *J*-INEPT experiment applied using a spin echo for chemical shift refocussing (blue and green contour lines) vs. COB-BIRD^{d,X} refocussing for ^{1}H decoupling over the indirect dimension (red and purple contour lines). The sequence has been applied to the $^{13}C_\alpha$ CH-groups of the 11-mer peptide shown on the righthand side (J). The 2D zooms of cross peaks (A - G) are shown with their amino acid label and the measured coupling. The COB-BIRD^{d,X} cross peaks have been shifted for visibility.

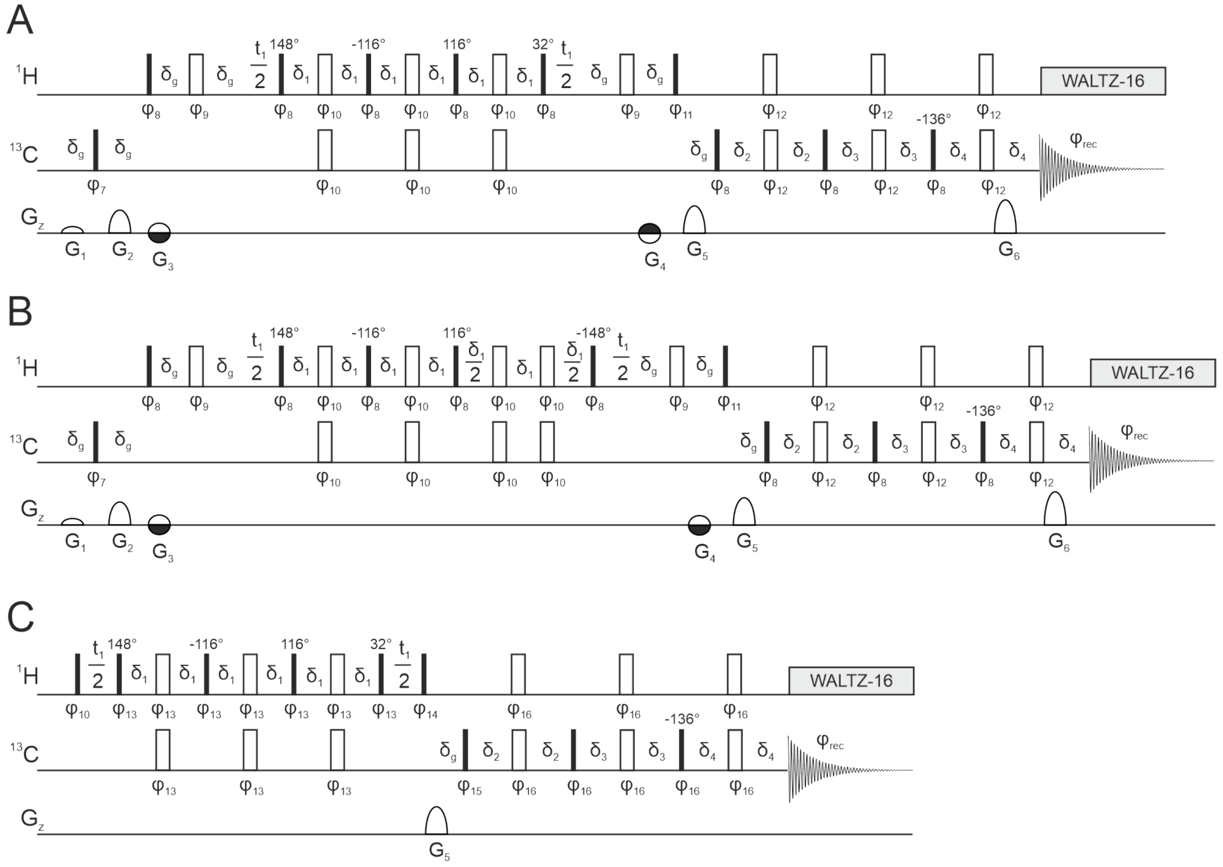

Fig. S14: **Full pulse sequences as applied in this publication.** Full pulse sequences of the ^{13}C-detected, ^{1}H-decoupled, COB3-refocussed experiments as applied in Figs. 6 (A), S9 (B), S10 (B), and S13 (C). Pulse sequences in A and B use echo-antiecho coherence selection. The phases can be cycled as ϕ_7 = x, y, -x, -y, ϕ_8 = x, ϕ_9 = x, -x, ϕ_{10} = x, x, -x, -x, ϕ_{11} = y, ϕ_{12} = x, -x, -x, x, ϕ_{13} = x, -x -x, x, -x, x, x, -x, ϕ_{14} = y, -y, ϕ_{15} = 8(x), 8(-x), ϕ_{16} = x, x, -x, -x, -x, -x, x, x, ϕ_{17} = 2(x, -x, -x, x), 2(-x, x, x, -x). Open rectangles represent 180° pulses, solid rectangles without flip angle indication represent 90° pulses and all other pulses have their respective flip angle indicated above. Delays correspond to δ_1 = 2.583 ms, δ_2 = 0.5401 ms, δ_3 = 1.065 ms, δ_4 = 1.0702 ms

Assignment	^{13}C T_1 [s]	^{13}C T_2 [s]	^{1}H T_1 [s]	^{1}H T_2 [s]
**2**	5.41	0.602	3.66	0.267
**4**	3.01	0.465	3.24	0.253
**5**	4.74	0.552	3.92	0.442
**6**	5.88	0.374	4.25	0.355
**8**	3.44	0.368	2.50	0.203
**9'**	2.49	0.174	2.07	0.192
**9"**			/	/
**10'**	2.37	0.381	2.01	0.184
**10"**			1.99	0.173
**11'**	2.10	0.301	1.85	0.175
**11"**			1.56	0.173
**12**	2.00	0.384	1.58	0.278

Table S4: **Relaxation times measured for 100 mM (-)-nicotine dissolved in a lyotropic 9% poly-γ-benzyl-L-glutamate/$CDCl_3$ mesophase at 600 MHz magnetic field strength.** Data is given according to the assignment used in Fig. 6 of the main text.

Assignment	$^{13}C\alpha$ T_1 [s]	$^{13}C\alpha$ T_2 [s]	$^{1}H\alpha$ T_1 [s]	$^{1}H\alpha$ T_2 [s]
Lys_1	0.526	0.244	2.02	0.212
Lys_2	0.435	0.276	1.84	0.192
Leu_3	0.476	0.223		
Phe_4	0.453	0.298	1.94	0.195
Lys_5				
Lys_6				
Ile_7	0.524	0.254	2.12	0.173
Leu_8	0.585	0.328	1.78a	0.175a
Lys_9				
Tyr_{10}	0.562	0.283	1.93	0.207
Leu_{11}	0.457	0.201	1.78a	0.175a

Table S5: **Relaxation times measured for the $^{13}C_\alpha$ and $^{1}H_\alpha$ of the 11-mer peptide measured in Fig. S13 at 850 MHz field strength.** Data is presented according to the assignment as used in Fig. 13.

8 Program for the simulation of ν_S- vs. J-dependence of BIRD sequences

Figures for the ν_S vs. J dependence of BIRD sequences were simulated using a self-written program. It has been programmed using JULIA version 1.9 with Visual Studio Code (VSCode) environment. JULIA can be downloaded and installed for free using the instructions given at `https://julialang.org` . The VSCode environment is one of several options.
